# Cortisol treatment impairs path integration and alters grid-like representations in the male human entorhinal cortex

**DOI:** 10.1371/journal.pbio.3003661

**Published:** 2026-03-12

**Authors:** Osman Akan, Varnan Chandreswaran, Henry D. Soldan, Anne Bierbrauer, Nikolai Axmacher, Oliver T. Wolf, Christian J. Merz

**Affiliations:** 1 Department of Cognitive Psychology, Institute of Cognitive Neuroscience, Faculty of Psychology, Ruhr University Bochum, Bochum, Germany; 2 Department of Neuropsychology, Institute of Cognitive Neuroscience, Faculty of Psychology, Ruhr University Bochum, Bochum, Germany; 3 Institute of Systems Neuroscience, Medical Center Hamburg-Eppendorf, Hamburg, Germany; Universitat Jaume 1, SPAIN

## Abstract

Acute stress triggers the release of cortisol, which broadly affects cognitive processes. Path integration, a specific navigational process, relies heavily on grid cells in the entorhinal cortex. The entorhinal cortex contains glucocorticoid receptors and is therefore likely to be influenced by cortisol, though little is known about this relationship. Given the role of the entorhinal cortex in neurological diseases such as Alzheimer’s Disease, investigating the effects of cortisol on this brain region may offer insights into how stress affects these diseases. In this study, we examined the effects of cortisol on human path integration in 39 healthy men across two sessions. On each day, they received either 20 mg cortisol or a placebo and performed a virtual homing task during functional magnetic resonance imaging (fMRI). Cortisol markedly impaired path integration performance, independent of incoming distance or the presence of spatial cues, but did not affect navigational pattern as measured by proximity to the landmark. fMRI results showed that cortisol increased the activation of right caudate nucleus in the presence of landmarks. Using a representational similarity analysis, we observed grid-like representations in the right entorhinal cortex specifically on day one under placebo, but these were diminished by cortisol. Grid-like representations were associated with PI performance dependent on the availability of spatial cues and cortisol administration, suggesting that cortisol may interfere with the typical relationship of grid cells and PI. Overall, the study indicates that cortisol-induced disruption in grid cell function in the entorhinal cortex may underly stress effects on path integration.

## Abstract 2

Stress impacts navigational performance and involves cortisol release, but how cortisol impacts brain functions supporting navigation remains unclear. This study in men shows that cortisol administration impairs path integration, a specific navigational process, and reduces grid-like brain activity patterns in the entorhinal cortex, offering new insights into the effects of stress on navigation.

## Introduction

Finding ourselves in situations where we lost orientation can easily invoke feelings of discomfort or stress, but we also often experience the inverse relationship of stress causing spatial disorientation, mainly through altering the way we navigate. For example, under time pressure, we tend to use more familiar and less accurate trajectories [1]. In general, the effects of stress on human spatial navigation remain poorly understood, despite navigation being strongly dependent on the hippocampus and adjacent cortical regions, which are primary targets of stress hormones. Investigating the effects of stress on navigation may ultimately deepen our understanding of neurological diseases, particularly since navigational deficits could serve as early biomarkers for Alzheimer’s Disease [2], and stress has been linked to both its pathogenesis and progression [3].

The stress response involves the activation of the rapid sympathetic–adrenal–medullary axis (SAM) and the slow hypothalamic–pituitary–adrenal axis (HPA), generally conceptualized as an adaptive reaction [4]. SAM axis activity initiates the release of catecholamines (noradrenaline and adrenaline), while HPA axis activity leads to the release of glucocorticoids (cortisol in humans, corticosterone in most rodents). Cortisol effects are mainly exerted by binding to mineralocorticoid receptors (MRs) and glucocorticoid receptors (GRs), while noradrenaline binds to adrenergic receptors [5,6]. Thereby, acute stress or pharmacological cortisol administration affect several cognitive domains including executive functions, episodic memory, fear conditioning, and decision-making [7–10].

During spatial navigation, different processes and strategies can be applied depending on several factors including environmental features [11]. One navigational process is path integration (PI), which is mainly used in absence of spatial cues and for short trajectories because error accumulation makes it less suited for longer trajectories. PI involves integration of self-referential information to estimate the current position and orientation in relation to an arbitrary reference point [12]. On the neuronal sheet, PI has been related to head-direction cells [13] and grid cell firing in the entorhinal cortex (EC; [14–17]). Specifically, the characteristic arrangement of grid cell firing fields in a regular hexagonal pattern [18,19] may provide a general spatial metric of distances [20]. In the presence of spatial cues, however, additional neural systems tuned to the specific type of cue (e.g., boundary or landmark) are recruited, including hippocampus [21] and posterior cingulate/retrosplenial cortex (PC/RSC; [22]). These additional systems may either stabilize grid cell firing [23] or support complementary navigational strategies. Because processing in the striatum, especially the caudate nucleus, is involved in landmark-based navigation [24] and appears to be enhanced under acute stress [25], the presence of spatial cues likely moderates stress hormone effects on either performance or strategy use during navigational processes.

Crucially, the entorhinal cortex is an important mediator of the stress response itself [26–29] and the abundance of GRs in the entorhinal cortex [30] as well as its interdependence with the hippocampus makes it a likely target of cortisol effects. Accordingly, acute stress impairs PI over long distances and in environments with little spatial information [31], and chronic stress is associated with impaired PI under similar conditions [32]. These effects might reflect cortisol-induced alterations in grid cell activity in the entorhinal cortex, potentially through a GR-mediated decrease of inhibitory signal in layer 2 of the medial entorhinal cortex [33]. Such a shift in the balance between excitation and inhibition is presumably affecting the functional output of grid cells, likely causing changes in navigation. Stress also alters navigational strategies, leading to less efficient routes and the avoidance of shortcuts [34,35]. Our recent work showed a stress-induced shift in navigational pattern, causing participants to opt for routes closer to a landmark [31]. This suggests an increased recruitment of landmark-based strategies under stress, possibly due to enhanced striatal processing.

**Fig 1:**
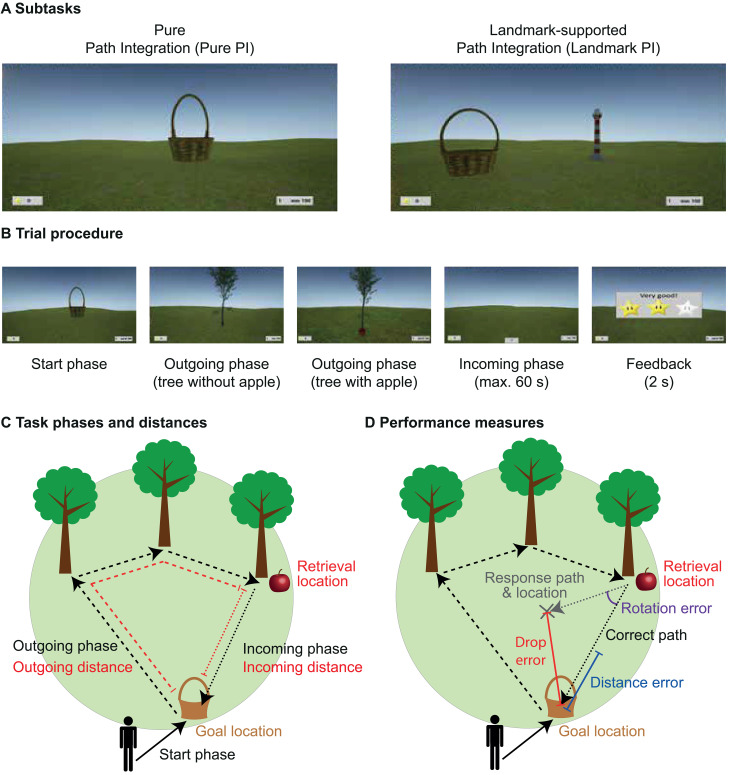
Experimental path integration task. **(A)** While the Pure PI subtask consisted only of a grassy plain, the Landmark PI subtask additionally contained a central lighthouse serving as spatial cue. **(B)** Each trial began with the “start phase”, where participants navigated to a basket (goal location), the location of which they should encode. In the following “outgoing phase,” they navigated to a variable number of trees (1–5) until reaching a tree containing an apple (retrieval location). Then, during the “incoming phase,” participants had to find the way back to the goal location before receiving feedback via zero to three stars according to performance based on the drop error (see D). Basket and trees disappeared as soon as they were reached. **(C)** Outgoing phase (dashed black line) and incoming phase (dotted black line) were quantified according to their spatial distances: outgoing distance corresponded to the cumulated distance from goal to retrieval location (dashed red line), and incoming distance to the Euclidean distance between retrieval and goal location (dotted red line). **(D)** General PI performance was assessed via the drop error, which corresponded to the distance between response location (marked with an X) and goal location (solid red line). The drop error can further be differentiated into distance error, referring to the difference between retrieval-to-goal distance and retrieval-to-response distance (blue line), and rotation error, depicting the angle between the retrieval-to-goal path and the retrieval-to-response path (purple arc). Figure adapted from Bierbrauer and colleagues [22] under CC BY 4.0.

Stress effects on human PI have so far been demonstrated only on the behavioral level. In this study, we aimed to investigate whether cortisol administration mimics the effects of stress on PI and to uncover the underlying neural mechanisms using functional magnetic resonance imaging (fMRI). This would enable us to bridge the gap between stress effects on behavioral PI performance and the impact of stress hormones on putatively underlying brain structures and neural processes. We hypothesized that cortisol impairs grid cell activity in the entorhinal cortex, and that this alteration constitutes a neural basis for navigational deficits. To assess PI performance, we used an adapted version of a virtual homing task (“Apple Game”) implemented by Bierbrauer and colleagues [22], in which participants are required to integrate across their outgoing trajectory to a goal and to calculate home-coming vectors based on visual cues only. In different subtasks of this virtual PI task, participants can either rely only on visual flow (Pure PI) or on visual flow and a central landmark (Landmark PI; Fig 1). To measure proxies of grid cell activity, we analyzed so called “grid-like representations” (GLRs; [22,36,37]).

Our results strongly support the hypothesis that stress-induced deficits of PI are mediated via cortisol, but they do not show that cortisol changes the navigational pattern towards the landmark. The fMRI data revealed (i) significant activation of the posterior cingulate and caudate nucleus during Landmark PI, and (ii) a selective engagement of the caudate nucleus during Landmark PI under cortisol administration. Additionally, they showed that the entorhinal cortex is most relevant during Pure PI under placebo. Importantly, our findings suggest that cortisol impairs GLRs.

## Results

**Fig 2:**
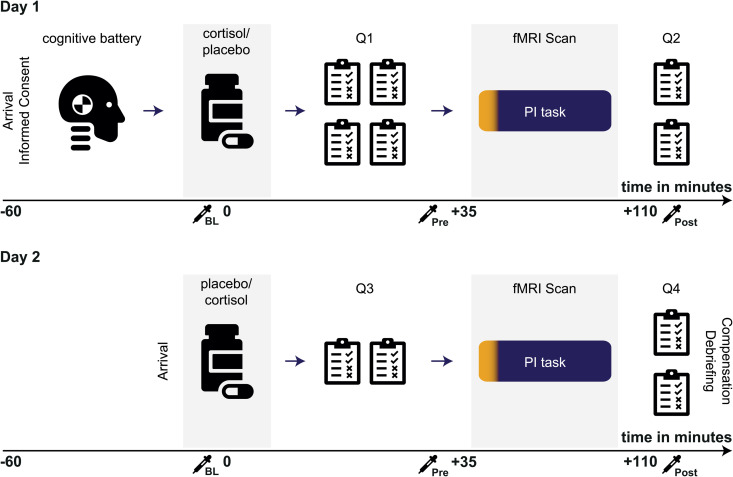
Experimental procedure. All participants were tested in both conditions (cortisol and placebo) on two testing sessions with an inter-session interval of one week. At the beginning of day one, participants underwent a cognitive test battery, which lasted about 40 min. Participants then received either 20 mg of cortisol, or a placebo. Afterwards, questionnaires (Q1) assessing demographic, psychological and medical data were filled out. Participants then were prepared for the fMRI scan, and the PI task started approximately 40 min after cortisol/placebo administration, before answering final questionnaires including a treatment guess and assessment of individual navigational strategies (Q2). One week later, participants were invited for the second testing session. The procedure on day two was similar, except that it did not contain a cognitive test battery, included less questionnaires and involved the respective other pharmacological intervention as compared to day one (crossover design). Numbers on x-axes reflect time-points relative to tablet administration, 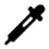 collection of saliva sample, PI: path integration.

We analyzed data from 39 men aged 19–34 years (24.18 ± 4.55 years; mean ± SD) participating in a two-day within-subject crossover design. Participants received either 20 mg of cortisol or visually identical placebo tablets on day one, and the respective other treatment on day two (one week later), before completing the Apple Game during fMRI recordings on both days. The order of administration was counterbalanced across participants (Fig 2; see also Materials and methods). To examine the effects of cortisol on PI, we conducted univariate and multivariate fMRI data analyses and built several linear mixed models (see S1 Table for an overview). In all mixed models, “subject” was added as random factor, and age, testing day, and sequence (to control for order effects) as covariates.

### Cortisol administration impairs PI performance irrespective of incoming distance and availability of spatial cues

The analysis regarding the success of cortisol administration revealed elevated cortisol concentrations under cortisol compared to placebo treatment for the entire PI task (S2 Table, see also Materials and methods). In the PI task, we assessed measures for incoming distance, PI performance and navigational pattern during the experimental task (see Materials and methods). *Incoming distance* refers to the Euclidean distance between retrieval and goal location (Fig 1C). Overall PI performance was assessed by the drop error, i.e., the Euclidean distance between response location and goal location (Fig 1D). For the navigational pattern analysis, to investigate the specific role of the landmark under cortisol, we measured the distance between the goal and the spatial cue (goal-to-landmark distance), and the mean Euclidean distance of the moving participant from the landmark across all time points of the incoming phase (movement-to-landmark distance).

**Fig 3:**
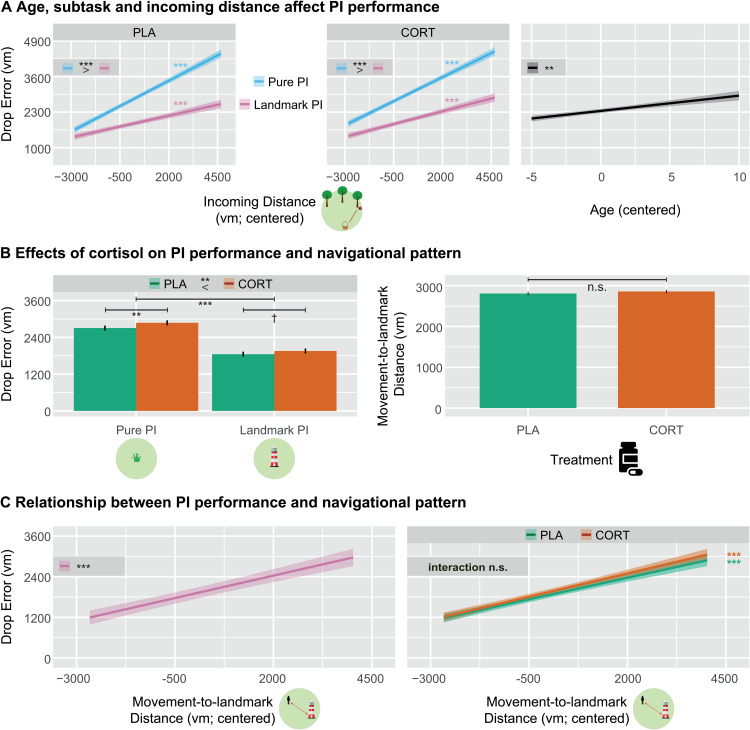
Predictors of PI performance and strategy use. **(A)** The effect of longer incoming distances leading to higher drop errors was more pronounced when no spatial cues were available (left), and older age led to higher drop errors (right). **(B)** CORT led to overall higher drop errors (left), but navigational pattern during landmark PI was preserved (right). **(C)** In Landmark PI, the movement distance to the landmark predicted performance, irrespective of treatment. Significant slopes are indicated via asterisks. Plots show estimated marginal means derived from linear mixed-effects models (see S2 Fig for extended plots). Error bars and confidence bands represent SEM. Pure PI: pure path integration, Landmark PI: landmark-supported path integration, CORT: cortisol, PLA: placebo, vm: virtual meters, n. s.: not significant, ***p < .001, **p < .01, *p < .05. The data underlying panel 3B can be found at https://osf.io/c8u57.

To investigate whether cortisol influences PI performance, we built a linear mixed model, in which we investigated effects of subtask (two levels: Pure PI versus Landmark PI), incoming distance, and treatment (two levels: cortisol versus placebo) on overall PI performance (i.e., drop error) on the level of single trials (see S3 Table for extended analyses). This analysis revealed similar findings as in our previous work [22,31,32]: We observed main effects of subtask (*F*_(1,4945)_ = 432.88, *p <* .001, *η*_p_^2^ = .080), and of incoming distance (*F*_(1,4945)_ = 364.99, *p <* .001, *η*_p_^2^ = .069), indicating higher errors in Pure PI than Landmark PI and for longer incoming distances, respectively. Besides, we found an interaction effect between subtask and incoming distance (*F*_(1,4949)_ = 44.99, *p <* .001, *η*_p_^2^ = .009; Fig 3A, left), indicating a stronger relationship between incoming distance and drop error in Pure PI as compared to Landmark PI (*t*_(4949)_ = 6.71, *p <* .001, *d* = 0.095). Moreover, we found a main effect of age (*F*_(1,36)_ = 12.23, *p =* .001, *η*_p_^2^ = .254; Fig 3A, right), reflec*t*ing higher drop errors in older age. No effects of testing day (*F*_(1,4945)_ = 0.39, *p =* .532, *η*_p_^2^ < .001) or sequence (*F*_(1,36)_ = 2.18, *p =* .148, *η*_p_^2^ = .057) emerged, indicating the absence of an order effect. Importantly, we found a main effect of treatment on drop error (*F*_(1,4945)_ = 10.46, *p =* .001, *η*_p_^2^ = .002; Fig 3B, left), indicating that cortisol impaired overall PI performance. Cortisol did not interact with other variables, suggesting a general effect independent of spatial cues or incoming distance.

In a second step, we built linear mixed models only including Landmark PI to examine the navigational pattern in the presence of the landmark under heightened cortisol levels. These models aimed at investigating the role of goal-to-landmark distance and its interaction with cortisol on PI performance, and at investigating whether cortisol affected the navigational pattern (irrespective of performance), respectively. In the first model, we found that a longer distance between goal location and landmark predicted worse PI performance, and cortisol did not moderate this effect (*F*_(1,2453)_ = 348.20, *p*<.001, *η*_p_^2^ = .124). Like in the main models, older age again significantly reduced PI performance (*F*_(1,36)_ = 6.30, *p* = .017, *η*_p_^2^ = .149). In the second model, we found no effect of treatment on movement distance to the landmark (*F*_(1,2455)_ = 1.37, *p*=.243, *η*_p_^2^ < .001; Fig 3B, right), showing that cortisol treatment had no effect on how closely participants navigate to the landmark.

The behavioral results so far have demonstrated that cortisol impairs PI performance, while the navigational pattern with respect to landmark proximity was preserved (if available). Generally, these variables may be connected, and we conducted a further analysis examining the effect of movement distance to the landmark on the drop error. Indeed, navigating further away from the landmark was associated with worse performance (*F*_(1,2453)_ = 106.37, *p* < .001, *η*_p_^2^ = .042; Fig 3C, left), and this effect was equally observed under placebo and under cortisol as evidenced by a lack of an interaction effect (*F*_(1,2459)_ = 0.16, *p* = .689, *η*_p_^2^ < .001; Fig 3C, right).

### Cortisol administration enhances activation in the right caudate nucleus in the presence of landmarks

We tested the contrasts “Landmark PI> Pure PI”, “CORT> PLA” and their interaction “(Landmark PI> Pure PI)_CORT_> (Landmark PI> Pure PI)_PLA_”—as well as the respective reverse contrasts—during the outgoing and incoming phases. We performed exploratory whole-brain analyses and region of interest (ROI) analyses in left and right hippocampus, left and right caudate nucleus, bilateral posterior cingulate, and left and right posterior-medial entorhinal cortex for each contrast. For the whole-brain analysis, statistical parametric maps were initially thresholded at a family-wise error (FWE)-corrected α level of *p* < 0.05 across the whole brain and clusters were considered significant at *p* < 0.05, FWE-corrected (extent threshold of five voxels). For ROI analyses, the significance threshold was set to *p* < 0.05 on voxel level, corrected for multiple testing within each ROI using FWE-correction with the small volume correction option of SPM12.

The exploratory whole-brain analyses revealed significantly higher activation in left superior parietal lobule, bilateral precuneus (adjacent to posterior cingulate) as well as in visual areas including left superior and right middle occipital gyri during Landmark PI compared to Pure PI, presumably reflecting the higher amount of visual information (see S3 Fig and S4 Table). No significant clusters were found on whole-brain level for the other contrasts.

**Fig 4:**
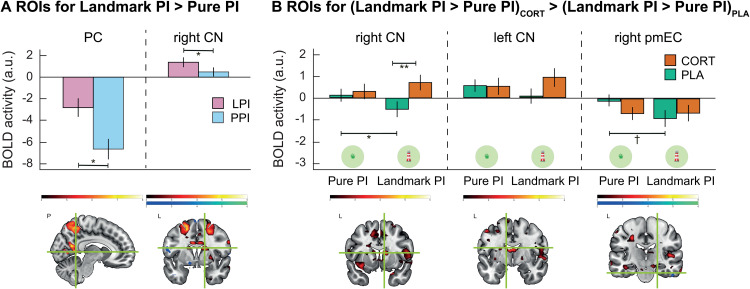
Univariate fMRI results. **(A)** Significantly higher activation in posterior cingulate and right caudate nucleus during Landmark PI as compared to Pure PI. **(B)** Significantly higher differentiation between subtasks under different treatments or between treatments in different subtasks in right caudate nucleus, left caudate nucleus and right posterior-medial entorhinal cortex. Activation shown for peak voxels of significant ROIs only. Error bars represent SEM. PI: path integration, CORT: cortisol, PLA: placebo, n.s.: not significant, ** *p* < .01, * *p* < .05, ^†^ *p* < .10. The data underlying this figure can be found at https://osf.io/c8u57.

The ROI analyses (see S4 Fig for overview of masks) showed higher activation of posterior cingulate (*t*_(35)_ = 6.33, *p*_FWE_ *<* .001) and right caudate nucleus (*t*_(35)_ = 4.45, *p*_FWE_ *=* .010) in Landmark PI compared *t*o Pure PI (*Landmark PI > Pure PI*; Fig 4A), while no significant differences were found for the other ROIs. Furthermore, no ROI showed differential activation in the contrast between cortisol and placebo (*CORT > PLA*). Importantly, however, we observed higher activation in the right posterior-medial entorhinal cortex (*t*_(35)_ = 2.78, *p*_FWE_ *=* .045), right caudate nucleus (*t*_(35)_ = 4.62, *p*_FWE_ *=* .006), and left caudate nucleus (*t*_(35)_ = 3.90, *p*_FWE_ *=* .035) in the interac*t*ion contrast of Landmark PI versus Pure PI after cortisol compared to placebo administration (*[Landmark PI > Pure PI]*_*CORT*_*> [Landmark PI > Pure PI]*_*PLA*_).

Pairwise post-hoc tests showed that these interactions reflected relatively higher activation of the right caudate nucleus (i) under cortisol compared to placebo during Landmark PI (*t*_(35)_ = 3.43, *p*_Bonferroni_ *=* .006, *d* = 0.550), and (ii) during Pure PI compared to Landmark PI under placebo (*t*_(35)_ = 2.82, *p*_Bonferroni_ *=* .032, *d* = 0.291), as well as a trend for a higher activation of the right posterior-medial entorhinal cortex during Pure PI than during Landmark PI under placebo (*t*_(35)_ = 2.45, *p*_Bonferroni_ *=* .077, *d* = 0.366). No significant pairwise comparisons emerged for the left caudate nucleus (all *t* ≤ 2.29, all *p*_Bonferroni_ ≥ .112, all *d* ≤ 0.360; Fig 4B). These findings indicate a reduced recruitment of the right caudate nucleus during Landmark PI compared to Pure PI under placebo, but enhanced recruitment during Landmark PI under cortisol compared to placebo, suggesting a relatively increased relevance of this region in the presence of landmarks under cortisol. Conversely, the findings suggest that the presence of landmarks can reduce recruitment of the right posterior-medial entorhinal cortex. No other ROI exhibited higher activation for this contrast, nor for any of the reverse contrasts.

On an exploratory level, we analyzed whether the neural differences between the subtasks were confounded by difficulty (S5 Fig). In this analysis, the contrast between Landmark PI and Pure PI was only significant in the posterior cingulate (*t*_(35)_ = 4.44, *p*_FWE_ *=* .029), while the right caudate was not significant any longer (*t*_(35)_ = 1.97, *p*_FWE_ *=* .705). The contribution of the posterior cingulate to landmark processing thus persists after controlling for total difficulty. On the other hand, right caudate nucleus appears to not be generally involved in landmark processing. However, when looking into the interaction with treatment, we observed a higher differentiation between the subtasks under different treatments for the caudate nucleus (both left: *t*_(35)_ = 3.96, *p*_FWE_ *=* .026, and right: *t*_(35)_ = 3.86, *p*_FWE_ *=* .034), resembling the results in the total sample. Moreover, the left hippocampus (*t*_(35)_ = 4.80, *p*_FWE_ *=* .004) also showed higher activation for the contrast between Landmark PI and Pure PI for different treatments, while the right posterior-medial entorhinal cortex did not any longer. This lack of an effect in the posterior-medial entorhinal cortex in the subsample could be related to reduced power due to the halved number of trials, particularly because this is a very small brain region.

Lastly, in an attempt to replicate the findings reported in Bierbrauer and colleagues [22], we conducted two additional analyses examining representations of integrated path and goal proximity (see S1 Text). These analyses partially replicated the original results (see S6 and S7 Figs).

### Cortisol administration impairs GLRs, which are associated with PI performance depending on subtask and treatment

To assess GLRs, we used a multivariate approach and focused on the right entorhinal cortex, consistent with previous studies [22,36]. Accordingly, due to their 6-fold rotational symmetry, grid cells should show more similar activity during movements along directions that differ by 60° (aligned movements) as compared to those differing by 30° (misaligned movements; see also Materials and methods).

**Fig 5:**
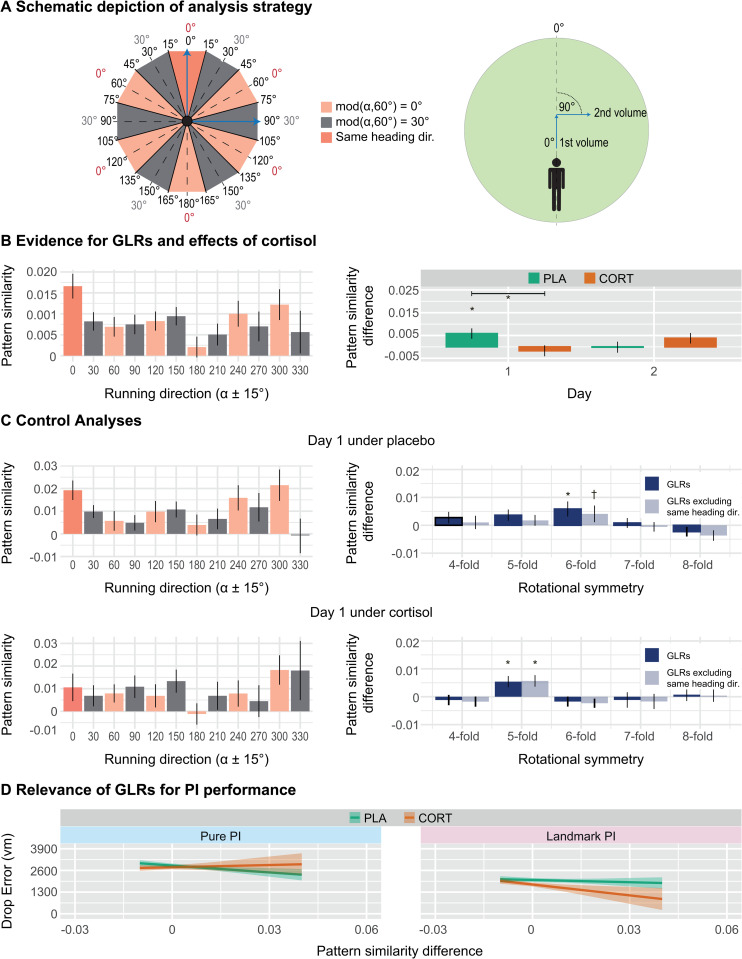
Cortisol effects on GLRs and their behavioral relevance. **(A)** Inner numbers represent angular differences in 360° space, whereas outer numbers show differences in 60° space (left panel). Higher pattern similarity was expected for angular differences of mod(*α*,60°) = 0° (rose; *α* as angular difference between two movement directions) than for angular differences of mod(*α*,60°) = 30° (gray). The same result was expected when discarding pattern similarities of movements for the same heading direction (dark rose). Two exemplary fMRI volumes representing different movement directions (blue arrows; right panel). In the first volume, the participant navigates at an angle of 0° with respect to the (arbitrary) reference axis, while navigating at an angle of 90° in the second volume. This results in an angular difference of 90° in 360° space, corresponding to 30° in 60° space and thus mod(*α*,60°) = 30° (compare blue arrows on the left panel). **(B)** Average pattern similarity over the entire time series of all voxels in the right entorhinal cortex for all directions of aligned (rose) and misaligned (gray) fast runs (left panel). Differences in GLRs (i.e., pattern similarity difference) depending on treatment and day (right panel). **(C)** Average pattern similarity (left panels) as well as control analyses (right panels) for treatment groups on day one. **(D)** Relevance of GLRs for PI considering day one. Error bars and confidence bands represent SEM. GLRs: grid-like representations, PI: path integration, CORT: cortisol, PLA: placebo, vm: virtual meters, n.s.: not significant, ** *p* < .01, * *p* < .05, ^†^ *p* < .10. The data underlying panels 5B and 5C can be found at https://osf.io/c8u57.

We first aimed to confirm the presence of GLRs by comparing activity during aligned versus misaligned movements (Fig 5A) and then tested whether they were affected by cortisol. Indeed, we found higher similarities between aligned movements (mod(*α*,60°) = 0°) than misaligned movements (mod(*α*,60°) = 30°) across all days and treatments, demonstrating the presence of GLRs in our study (*t*_(71)_ = 1.75, *p =* .042, Fig 5B, left). The permutation test further strengthened the robustness of this finding, as the obtained z-scores were significantly different from zero on group level (*t*_(71)_ = 1.69, *p =* .048). Subsequent analyses revealed no main effect of treatment on GLRs (*F*_(1,67)_ = 0.61, *p* = .437, *η*_p_^2^ = .009), but we observed a significant treatment by day interaction (*F*_(1,67)_ = 5.99, *p* = .017, *η*_p_^2^ = .082; Fig 5B, right). Due to the crossover design, the interaction cannot easily be interpreted, as it may also include order effects. We thus decided to examine differences between groups on day one [38]. When doing this, post-hoc tests revealed a significant effect of treatment (*t*_(67)_ = 2.28, *p =* .026, *d* = 0.761), indicating higher GLRs during placebo than during cortisol (whereas analogous univariate contrast analyses restricted to day one, both whole-brain and ROI-based, did not reveal any brain region with significantly higher activation). Indeed, separate analyses confirmed the presence of GLRs under placebo (*t*_(18)_ = 2.16, *p*_Šídák_ *=* .044) but not under cortisol treatment (*t*_(16)_ = −0.97, *p*_Šídák_ *=* .826). Control analyses of GLRs from day one under placebo (Fig 5C, top) confirmed that pattern similarity differed between aligned and misaligned movements only for 6-fold rotational symmetry (*t*_(18)_ = 2.16, *p =* .022), but not for 4-fold, 5-fold, 7-fold, or 8-fold rotational symmetries (all *t* ≤ 1.81, all *p*_*Bonferroni*_ ≥ .175), and removing movements during same heading directions (±15° from 0°) preserved (by trend) 6-fold rotational symmetry and thus GLRs (*t*_(18)_ = 1.37, *p =* .093). In contrast, under cortisol, the control analyses showed no evidence for 6-fold symmetry (Fig 5C, bottom). Although potentially confounded by order effects and thus difficult to interpret, GLRs were notably not found on day two under either treatment.

Then, we investigated whether GLRs were behaviorally relevant using a linear mixed model incorporating effects of subtask, incoming distance, and GLRs. Because of the interaction effect between day and treatment, we again only focused on day one. We found similar effects as in the purely behavioral model (main effects of subtask, incoming distance, age, and the subtask by incoming distance interaction) with the addition of an interaction between subtask, treatment and incoming distance (*F*_(1,2264)_ = 4.33, *p* = .037, *η*_p_^2^ = .002), and an interaction between subtask, treatment, and GLRs (*F*_(1,2256)_ = 5.48, *p* = .019, *η*_p_^2^ = .002; Fig 5D). For the former effect, post-hoc analyses revealed that the relationship between incoming distance and drop error in pure PI is stronger under placebo than under cortisol (*t*_(2263)_ = 3.05, *p*_Šídák_ *=* .014), indicating a higher importance of incoming distance for performance under placebo. In contrast, post-hoc analyses for the latter interaction failed to reach significance, but a visual inspection of this finding suggested that GLRs are generally positively associated with PI performance, except under cortisol in the Pure PI subtask, which showed the inverse relationship.

Lastly, we examined whether the separate analysis of GLRs in the Pure PI and Landmark PI conditions revealed further insights. This analysis showed a largely similar pattern as in the general analysis (see S8 Fig), and further suggested that GLRs may be more relevant under Pure PI than under Landmark PI.

### Exploratory parametric modulation analysis of PI performance

We further conducted a parametric modulation analysis using a general linear model (GLM), including trial-wise inverted drop error values as regressor for the combined outgoing and incoming phases. We used separate parametric regressors for Landmark PI and Pure PI trials, estimating each regressor’s average effect against baseline across treatments as well as the contrast between treatments. In addition, we computed the contrast between LPI and PPI trials across and between treatments. These effects were tested exploratorily across the whole brain as well as in ROI analyses.

The whole-brain analyses revealed one cluster showing modulation across treatments on Landmark PI trials as well as three clusters showing modulation across treatments on Pure PI trials, while no differences were found between treatments (S9 Fig and S5 Table). For the contrast between subtasks (Landmark PI versus Pure PI), one cluster showed significantly stronger positive modulation on Landmark PI compared to Pure PI trials across treatments, while there were again no differences between treatments (S9 Fig and S5 Table). The ROI analyses did not reveal any significant modulation of the BOLD signal for any of the contrasts (all *p*_FDR_ > .983; S9 Fig).

## Discussion

We investigated whether cortisol administration affects PI performance, navigational pattern in the presence of a landmark, and associated brain activations in healthy young participants. We found that cortisol administration impaired overall PI performance, while preserving the navigational pattern with respect to landmark proximity. The univariate fMRI data analysis indicated a higher activation of the posterior cingulate and the right caudate nucleus when PI was supported by landmarks, and a selective engagement of the right caudate nucleus in this condition under cortisol. In the absence of landmarks, when participants could solely rely on optic flow, right posterior-medial entorhinal cortex showed relatively higher activation under placebo compared to cortisol. The multivariate fMRI data analysis showed the presence of GLRs in the right entorhinal cortex under placebo on day one, but not under cortisol, and their association with PI performance was based on subtask and treatment.

Our behavioral results indicated that cortisol impairs overall PI performance, an effect mediated by slight impairments of distance and rotation estimations (see S10 Fig). Even though we expected a detrimental cortisol effect, we assumed it to be more pronounced in environments with no spatial cues (Pure PI) and in trials with longer incoming distances, because we had previously found acute stress effects to be moderated by these variables [31]. Cortisol is a relevant mediator of stress effects and known to influence cognitive processes [8], but since its increase is only one part of the several biological processes involved in the stress response, effects of cortisol and acute stress are often not identical and sometimes even opposing [39].

An important principle of glucocorticoid effects is dose-dependency [40], referring to differential effects on neurons in a concentration-depending manner. Dose-dependency interacts with the local distribution pattern of MRs and GRs, which exhibit differences in their affinity to cortisol [5]. While MRs show greater affinity and are highly expressed in limbic structures, GRs possess lower affinity and are more uniformly distributed across the brain, with high abundance in the entorhinal cortex [30]. Thus, high cortisol concentrations like in the current study (much higher than average acute stress responses) may affect GRs in entorhinal cortex more strongly, behaviorally manifesting in reduced PI irrespective of moderators like environmental scarcity and incoming distance. In another previous study, we did not find a main effect of chronically elevated cortisol levels on PI performance, but subtle impairments of distance estimations under conditions of spatial scarcity and long incoming distance [32]. Again, cortisol concentrations were in a low-to-mid range, potentially being insufficient to occupy enough GRs to impair PI performance generally. We therefore propose that cortisol affects PI in a concentration-dependent manner, where lower concentrations lead to subtle effects, detectable in the absence of spatial cues and over long incoming distances, and where higher concentrations induce severe effects, detectable irrespective of environmental properties or incoming distance.

One seemingly surprising finding comprised the significant effect of age on PI performance. Even though it is well known that navigational ability decreases across the life span [41], functional PI deficits have been shown at considerably older age [42]. As our sample consisted of young adults, the question arises whether specific navigational processes, like PI, could even be affected earlier, potentially under specific circumstances. Indeed, Kunz and colleagues [21] showed that—even in a sample of 18–30 years old adults—spatial memory performance negatively correlated with older age. This may be related to higher alertness in younger age [43] which can be pronounced even within the range of 18–35 years old adults, or other factors like motivation. However, to more thoroughly investigate this question, future work specifically addressing this topic is warranted.

The role of landmarks during PI is to stabilize grid cell firing [23,44], thereby improving performance in general, an effect that is in accordance with our results. When examining the navigational pattern during Landmark PI irrespective of performance, we found that cortisol had no effect on how closely participants navigated to the landmark. In a previous study, we had shown that acute stress (on trend) changes the navigational pattern towards the landmark, and we argued that this change might reflect stress-induced enhanced use of landmark information, stronger recruitment of the posterior cingulate/retrosplenial cortex, or a shift towards striatal processing after acute stress [31]. While the fMRI data of our current study does not support the idea of higher recruitment of posterior cingulate/retrosplenial cortex, we indeed found higher activation in the right caudate nucleus under cortisol during Landmark PI compared to Pure PI, supporting the claim of increased striatal processing. Mechanistically, acute stress enhances the use of landmark information in the striatum via cortisol binding on MRs [45], especially in participants showing high cortisol responses. Thus, we would here expect converging effects of acute stress and cortisol, even though dose-dependency might again play a relevant role. With high cortisol concentrations, like in our current study, not only MRs are activated in the striatum, but also GRs, changing the relative activation of MRs and GRs and thereby altering neuronal processing, an idea referred to as *MR/GR ratio hypothesis* [46]. However, while the potential concomitant activation of both receptor types in this study might have contributed to the inconsistency in findings, our task is not specifically designed for investigating landmark-based strategies and their neuronal correlates, making it less comparable to studies targeting this phenomenon explicitly. Further research is needed to clarify the role of cortisol (concentration) on landmark use, both in general and specifically during PI.

The exploratory whole-brain analyses demonstrated higher activation in the left superior parietal lobule, bilateral precuneus and in visual areas including left superior and right middle occipital gyri in the presence of landmarks. The superior parietal lobe is critical for representing the body in space and for guiding movements based on spatial information [47]. During Landmark PI, it may support spatial attention (directing attention to the landmark and monitoring its position in relation to the self), spatial mapping (constructing and maintaining a coherent map incorporating the landmark), and sensorimotor integration (integrating self-motion information with the visual information from the landmark). The precuneus provides spatial information to hippocampus and entorhinal cortex [48], and integrates self-motion information within the environment [49]. During Landmark PI, the precuneus likely integrates landmark information with internal representations to continuously update the mental map, along with supporting processes of visuospatial imagery and attentional shifting. The superior and middle occipital gyri are involved in processing visual motion, object recognition and spatial aspects of visual stimuli [50,51]. During Landmark PI, their involvement is presumably related to the increased visual input and processing, namely satisfying the additional needs of identifying and localizing the landmark, processing visual motion including optic flow, and encoding spatial relationships. In summary, the higher activation of these areas during Landmark PI presumably reflects the additional cognitive and perceptual demands of processing and integrating visual landmark information into the navigation process, to enhance the accuracy and efficiency of PI. Additionally, the parametric modulation analysis of the drop error revealed that some of these brain regions (i.e., left inferior and left middle occipital lobule) were also related to PI performance.

The ROI analyses did not reveal any region that is modulated by PI performance, but showed less deactivation of the posterior cingulate and higher activation of the right caudate nucleus during Landmark PI, supporting the hypothesis of their crucial involvement in landmark processing [22,44,52,53]. Furthermore, we did not observe any ROI that shows generally increased activation under cortisol than placebo, but when incorporating subtask, we found effects in the right caudate nucleus and the right posterior-medial entorhinal cortex. The right caudate nucleus showed higher activation under cortisol during Landmark PI, suggesting that striatal processing might not be generally enhanced under stress, but specifically when usage of stimulus-response type of strategies (landmark processing in this case) is possible. Under placebo, the right posterior-medial entorhinal cortex showed less deactivation during Pure PI (on trend), highlighting the relevance of this region in the absence of spatial cues and suggesting that adding a landmark reduces its involvement. This is in accordance with the idea that additional brain regions contribute to PI when spatial cues become available [21,23].

The analysis of the subsample including difficulty-matched subtasks added evidence for the more general contribution of the posterior cingulate to landmark processing, while the caudate nucleus appears to be particularly related to landmark processing under cortisol. The absence of an effect in the posterior-medial entorhinal cortex may hint to a role of this area in high-distance pure PI conditions or could be related to reduced power. The addition of the left hippocampus, on the other hand, may indicate that this region is also relevant for the differentiation of subtasks under cortisol, even though the pattern of results remains unclear.

In the right entorhinal cortex, we further demonstrated the existence of overall GLRs. To control for potential order effects in our crossover design, we focused on differences between treatments on day one only. There, we found GLRs under placebo, but not under cortisol treatment. This finding is in accordance with the hypothesis that cortisol affects the entorhinal cortex and impairs GLRs, which may account for the PI deficits that we found in a series of studies including the current one [31,32]. More specifically, because cortisol administration impairs inhibitory transmission in layer II of the entorhinal cortex [33], we propose that cortisol-induced inhibition of grid cells in the medial entorhinal cortex causes behavioral PI deficits. Grid cells, and the medial entorhinal cortex, are generally related to PI performance, and lesions in the medial entorhinal cortex impair PI in rats [54,55], presumably related to impaired distance estimations [56]. Our analyses showed a relationship between GLRs and PI performance that depended on treatment and subtask. Even though post-hoc tests were not significant, visual inspection of the significant interaction showed inverted patterns for Pure PI and Landmark PI, suggesting that GLRs are positively associated with Pure PI performance under placebo, and under cortisol only in the presence of landmarks, while they are associated with reduced performance under cortisol in Pure PI. Based on previous studies [17,22,57], we assumed that GLRs should facilitate performance specifically in environments with no spatial cues, and while our secondary analysis suggested that GLRs might be more pronounced in Pure PI, our findings indicate that they could rather be generally correlated with better performance (independent of the subtask), except if there is a disturbance in normal functioning. In our case, this disturbance was the administration of cortisol, which appeared to interfere with performance particularly in the Pure PI condition, i.e., in environments without spatial cues, an idea that we postulated previously [31,32]. However, as already mentioned, this interpretation is at least partially speculative, because post-hoc effects were not significant, and further work is needed to clarify this relationship.

A few limitations of this study need to be addressed. First, due to increased power, we used a within-subject crossover design. This can lead to results where treatment and order effects cannot be disentangled, which we accounted for at the price of reducing sample size for GLR analyses by excluding data from day two. GLRs were notably not found on day two under either treatment, which might be due to the potential confound by order effects. We cannot definitively determine the extent to which these order effects contribute to the overarching interaction. Also, stress effects in within-subject tasks can differ from those in between-subject designs [58], even though we bypassed this issue by considering day one only (making it a pseudo between-subject design) for the cortisol effect on GLRs. Second, cognitive fatigue can water down stress hormone effects on cognition [39,58], and thus, the cognitive battery implemented before the task and the long duration of the task might have influenced our results, suggesting that cortisol effects on GLRs and PI could be even stronger than what we reported here. In addition, the Pure PI condition lacks stable orientation cues, making it difficult to determine whether cortisol disrupts grid coding itself or merely affects the consistency of grid orientation across trials. It could be the case that grid coding is present, but that cortisol impairs the consistency of this coding from one trial to the next. Last, our sample consisted of young healthy men, which is not representative for the entire population, because sex hormones play a major in moderating effects of stress and cortisol [59]. However, we have shown cortisol effects on PI in an older, female sample [32], which suggests that behavioral effects of stress might be similar between sexes, while the investigation of neuronal effects is warranted in more representative samples including women in future studies.

### Conclusions

Our current findings provide essential information about the underlying mechanisms of stress effects on PI, supporting the hypothesis that they are strongly mediated by effects of cortisol on the entorhinal cortex. Cortisol impaired overall PI performance in young healthy men, while the navigational pattern with respect to landmark proximity was preserved. The fMRI data further indicated that cortisol administration increased activation in the right caudate nucleus in the presence of landmarks. Lastly, we demonstrated GLRs in the right entorhinal cortex, which were associated with PI performance in interaction with the spatial environment and the administration of cortisol.

## Materials and methods

### Ethics statement

All participants were informed about the study procedures and provided written informed consent before the study began. The research was conducted in accordance with the Declaration of Helsinki and approved by the local ethics committee (Ethics Committee of the Medical Faculty, Ruhr University Bochum, approval no. 18-6368).

### Participants

We used a two-day within-subject crossover design and recruited 42 healthy men, two of which had to be excluded due to technical failures of the joystick and another one due to unsuccessful experimental manipulation (no cortisol increase), leaving a final sample size of *n* = 39, aged 19–34 years (24.18 ± 4.55 years; mean ± SD) and with a body mass index ranging between 18 and 29 kg/m^2^ (23.95 ± 2.54 kg/m^2^; mean ± SD). We recruited participants through online advertisements in social media networks, mailing lists and in university classes at Ruhr University Bochum. Exclusion criteria comprised an acute or history of disease (i.e., neurological, psychiatric, cardiovascular, immunologic), current or history of medical or psychological treatment, drug use, female sex, previous experience with the PI paradigm (see Experimental task), or any contraindication for participation in MRI studies. We only included men because of the well-known influence of sex hormones on the secretion of stress hormones [60] and the related changes in neural functioning [61]. All participants had normal or corrected-to-normal vision and received a compensation of 10€/hour (60–70€ in total) or course credits.

### Experimental design

Sample size was based on previous studies investigating PI [17], the functional relevance of GLRs [21,22], and effects of cortisol administration [62]. In addition, we conducted a power analysis for a repeated measures analysis of variance (rANOVA) with two conditions (placebo versus cortisol), *α* = 0.05, power (1 − *β*) = 0.8, and a medium effect size (*f* = 0.25). This analysis suggested a minimum of 34 participants. While this analysis does not exactly match our linear mixed-effects modeling approach, it provides a reasonable benchmark.

Participants received two tablets of 10 mg cortisol (hydrocortisone, Hoechst) on one testing day, and two visually identical placebos on the other testing day in a double-blind two-day within-subject crossover design. Order of conditions was counterbalanced across participants. The dosage of 20 mg was chosen in accordance with previous studies demonstrating effects of cortisol on behavioral and neuronal responses with similar dosages [63–67]. We assessed salivary cortisol using Salivettes (Sarstedt, Nümbrecht, Germany) collected at several time-points (see Fig 2). Saliva samples were stored at −20 °C until assayed. Salivary cortisol concentrations were extracted from the samples using a time-resolved fluorescence immunoassay (IBL, Hamburg, Germany) at the local biochemical laboratory of Ruhr University Bochum and are reported in nanomole per liter (nmol/l). Intra- and inter-assay coefficients of variations were below 6.1%.

### Experimental task

The Apple Game is a virtual PI task that was implemented by Bierbrauer and colleagues [22] via Unreal Engine 4 (Epic Games, version 4.11; see also Fig 1 and S1 Fig). In this task, PI is considered as the ability to integrate across several paths and to calculate home-coming vectors based on visual cues only. A circular arena formed by an endless grassy plain with a blue sky rendered at infinity, with a diameter of 13,576 virtual meters (vm), built the environment. Briefly, each trial was composed of three phases. In the “start phase”, participants first moved to a basket and memorized its location (goal location). Then, the “outgoing phase” followed, in which they navigated to a variable number of trees (1–5), which appeared consecutively in different locations, until a tree containing a red apple (retrieval location) was reached. The variable number of trees allowed manipulating the path of the outgoing phase and thus difficulty. Basket and trees disappeared upon arrival at the respective locations. During the “incoming phase”, participants lastly were asked to take the shortest path from the retrieval location back to the goal location. They pressed a button when arriving at the presumed location (response location) and received visual feedback via zero to three stars corresponding to the Euclidean distance between response location and goal location (drop error; three stars for <1,600 vm, two stars for <3,200 vm, one star for <6,400 vm). To investigate the influence of spatial cues on PI, the original version of the task contained three subtasks, of which we used two in this study. The “Pure PI” subtask was composed only of a grassy plain, forcing participants to solely rely on visual flow, while the “landmark-supported PI” (Landmark PI) subtask contained a central lighthouse as spatial cue. Participants first played up to eight training trials to get familiar with the task, during which a structural MRI scan (~6 min) was obtained. Then, a total of 64 experimental trials (32 per subtask) divided into four blocks of 16 trials were presented, during which functional MRI scans were assessed. The outgoing phase of the 32 trials of each subtask was composed of six trials with 1, 2, 4, and 5 trees, respectively, and eight trials with three trees in randomized order. Before every new trial, a fixation cross was presented with a variable duration 5–7.5 s (randomly distributed).

### Experimental procedure

Testing sessions were performed after 1 PM to avoid interference with the cortisol awakening response [68]. On day one, upon arrival, participants read study information and gave written informed consent, before undergoing a cognitive test battery, after which the pharmacological intervention and a series of questionnaires followed (see S6 Table). About 30 min after pharmacological intervention, participants were prepared for the fMRI session, during which the experimental PI task was conducted. Lastly, participants answered a final set of questionnaires. The testing session on day two followed one week later, and the procedure was essentially the same, except for the absence of a cognitive test battery and the respective other pharmacological intervention. Finally, participants were debriefed and compensated (see Fig 2).

### Behavioral data

To extract Apple Game data from computer-generated log-files we used MATLAB (2021b, The MathWorks, Massachusetts, US), including the Parallel Computing Toolbox (v6.12) and the CircStat Toolbox [69]. Statistical analyses were conducted in R [70] using the lme4 [71], lmerTest [72], and emmeans [73] packages.

### Behavioral data analysis

One participant did not show any cortisol increase after cortisol administration and thus was excluded from statistical analyses, leading to a sample size of *n* = 39 for behavioral analyses. We assessed several measures for path distance, PI performance and navigational pattern during the experimental task. For path distance, two parametrical measures were assessed: O*utgoing distance* represents the accumulated distance from the goal to the retrieval location, while *incoming distance* refers to the Euclidean distance between retrieval and goal location (Fig 1C). These measures represent different subcomponents of PI: Outgoing distance is relevant for keeping track of the traveled path in relation to the starting point (i.e., the later goal location), and incoming distance for calculating a direct vector to this goal location. Because previous studies indicated incoming distance to be more closely related to entorhinal cortex activation [52,74], and the results of our previous work supported this view [31,32], we again concentrated on this measure as a proxy of path distance. Moreover, overall PI performance is represented by the drop error, i.e., the Euclidean distance between response location and goal location (Fig 1D). Lastly, to investigate the specific role of the landmark under heightened cortisol levels, we assessed two variables only applicable in Landmark PI. The first of these reflects the distance between the goal and the spatial cue (goal-to-landmark distance), while the other represents the mean Euclidean distance of the moving participant from the landmark across all time points of the incoming phase (movement-to-landmark distance).

### MRI data

We acquired MRI data during the whole experimental task at the Bergmannsheil hospital in Bochum using a 3T Philips Achieva Scanner (X-Series, the Netherlands) with a 32-channel head coil. High-resolution whole-brain structural brain scans were acquired using a T1-weighted sequence with a 1 mm isotropic resolution, an FOV of 240 mm × 240 mm, and 220 transversally oriented slices during a total acquisition time (TA) of six minutes and three seconds. Blood oxygenation level-dependent (BOLD) contrast images were registered with a T2*-weighted gradient echoplanar imaging sequence with 2.5 mm isotropic resolution, TR = 2,500 ms, TE = 30 ms, FA = 85°, FOV = 96 mm × 96 mm, 45 transversal slices in ascending order without slice gap, and TA = 17.32 ± 2.01 mins (mean ± SD), corresponding to 415.67 ± 48.27 (mean ± SD) volumes. Variation in TA emerged because the task was self-paced, leading to different durations of runs. In addition to three dummy scans preceding data acquisition, the first five images of each session were discarded to allow for signal steady-state transition. We further used a shim box of 60 mm × 60 mm × 60 mm around the medial temporal lobe to increase temporal signal-to-noise ratio. The virtual environment was presented to participants via MR-compatible liquid crystal display goggles (VisuaStim Digital, Resonance Technology, Northridge, CA, USA) with a resolution of 800 × 600 pixels, and they navigated within the virtual environment using an MR-compatible joystick (Nata Technologies, Coquitlam, Canada).

fMRI data were preprocessed and subsequently analyzed using SPM12 implemented in MATLAB (2021a, The MathWorks, Massachusetts, US) and nilearn for Python (Nilearn contributors, 2025). Preprocessing included slice time correction and spatial realignment. For whole-brain and ROI analysis, fMRI scans were further normalized to MNI space using parameters from the normalization procedure of the segmented structural T1 image. Further, we applied spatial smoothing with a 5 mm isotropic Gaussian kernel to the normalized fMRI data.

We used masks of the left and right hippocampus, left and right caudate nucleus, and bilateral posterior cingulate (maximum probability masks; probability threshold: 0.25; Harvard-Oxford Cortical and Subcortical Structural Atlases, Harvard Center for Morphometric Analysis; https://cma.mgh.harvard.edu/). Because the medial entorhinal cortex is especially related to grid-cell activity in rodents and the human entorhinal cortex consists of structurally and functionally distinct subparts, where the anterior-lateral entorhinal cortex and the posterior-medial entorhinal cortex represent the homolog of rodent lateral and medial entorhinal cortex, respectively, we focused our univariate analysis on left and right posterior-medial entorhinal cortex using masks created by Maass and colleagues [75]. For the multivariate analyses, we performed automated segmentation of the entire right entorhinal cortex using the Automatic Segmentation of Hippocampal Subfields (ASHS) software with the ASHS-PMC-T1 Atlas [76,77].

### fMRI data analysis

We excluded three participants, whose entorhinal cortices were only partly covered, most likely due to susceptibility artifacts and other distortions, which are very strong in the medial temporal lobe [78], leaving a final sample size of *n* = 36 for the fMRI data analysis. In the first GLM, we modeled start phase, outgoing phase, incoming phase, and feedback separately for each of the two subtasks and each of the two treatments (16 regressors in total). The duration of these regressors corresponded to the duration of the respective phases in each trial. We tested three contrasts (plus their respective reverse contrasts): “Landmark PI> Pure PI”, “CORT> PLA” and their interaction “(Landmark PI> Pure PI)_CORT_> (Landmark PI> Pure PI)_PLA_”, specifically during the outgoing and incoming phases, which represent the actual PI task. All regressors were convolved with the hemodynamic response function before entering the GLM. As nuisance regressors, we included motion parameters as estimated in the realignment procedure and a high-pass filter (time constant = 128 s) was implemented. We performed whole-brain analyses on each contrast. Contrast images from the first-level analysis of each participant were entered into a GLM. Statistical parametric maps were initially thresholded at a FWE-corrected α level of *p* < 0.05 across the whole brain. We considered clusters significant at *p* < 0.05, FWE-corrected (extent threshold of five voxels). For all significant clusters, we provide maximum probability tissue labels with MNI coordinates derived from the Neuromorphometrics atlas as implemented in SPM12 (www.oasis-brains.org/; http://neuromorphometrics.com/). For ROI analyses, the significance threshold was set to *p* < 0.05 on voxel level, corrected for multiple testing within each ROI (FWE-corrected; using the small volume correction option of SPM12).

On an exploratory level, we re-ran the first GLM with matched task difficulty between subtasks. Disentangling task difficulty from the effects of spatial cues is inherently challenging, as difficulty varies across multiple levels and partially overlaps with cue availability: spatial cues facilitate navigation by enabling additional cognitive strategies, while difficulty can also be manipulated via path distance or cumulative angular rotation. To approximate matching for difficulty, we compared trials with high incoming distances in Landmark PI to trials with low incoming distances in Pure PI, matching overall performance levels rather than specific subprocesses. Consequently, residual differences in difficulty between subtasks cannot be fully excluded. To address this, univariate analyses were repeated in a subsample of trials, in which, within each participant, trials were ranked by incoming distance and the upper half of Pure PI trials and the lower half of Landmark PI trials were excluded. This substantially reduced performance differences between subtasks, although a small difference remained, which was accepted to preserve statistical power.

In a second GLM, we conducted an exploratory parametric modulation analysis of the drop error, which for each of the two subtasks included an unmodulated trial regressor as well as a parametric modulation regressor for trial-wise drop error modeling the combined outgoing and incoming phases of each trial (four regressors in total). The onsets of these regressors corresponded to the onset of the outgoing phase and the modeled durations corresponded to the added total durations of the outgoing and incoming phases of each trial. Drop error values were symmetrically inverted so that higher values of the parametric regressor would reflect better task performance. In addition, drop error values were normalized to range between 0 and 1 and mean-centered. All regressors were convolved with the hemodynamic response function before entering the model. We included motion parameters resulting from realignment of functional images during preprocessing as nuisance regressors. Additionally, we used a cosine model with a high-pass frequency of 0.01 Hz to account for slow temporal drifts in the signal. For each participant and session, beta maps were computed for the contrast of each subtask’s drop-error parametric modulation against baseline, and for the contrast between subtasks’ parametric modulations (Landmark PI versus Pure PI). We performed whole-brain analyses on the first-level drop error parametric modulation contrast images, computing two different second-level contrasts for each while accounting for subject-specific effects: The average first-level effect across treatments as well as the difference in first-level effects between cortisol and placebo. Resulting statistical parametric maps were thresholded at a false discovery rate (FDR)-corrected α level of *p* < 0.01 across the whole brain. Clusters were considered significant at *p* < 0.01, FDR-corrected and extending a minimum cluster size of 10 voxels. For all significant clusters, maximum probability tissue labels are reported based on the peak voxel’s MNI coordinates and segmentations derived from the Harvard-Oxford atlas as implemented in nilearn. For the ROI analysis of parametric modulation by drop error, *p* values were corrected for the number of repeated tests (number of ROIs and number of contrasts) using FDR-correction at *α* < 0.01.

To assess GLRs during fMRI, we performed a representational similarity analysis (RSA), using an approach analogous to previous studies [22,36]. This method assumes that grid cells in the entorhinal cortex, due to their 6-fold rotational symmetry, show similar activity patterns during movements that differ by 60° in angular space. Activity patterns should thus show higher similarity for movements with an offset of n*60°, where *n* = {0, 1,..., 6}, and lower similarity for movements with an offset of n*60° + 30°, where *n* = {0, 1,..., 5}. We refer to the first condition as “mod(*α*,60°) = 0°” and to the second condition as “mod(*α*,60°) = 30°” (where *α* represents the angular difference between movement directions, and “mod” denotes the modulo operator; see Fig 5A). For the analysis we used the preprocessed fMRI data. To account for the hemodynamic response, BOLD time series were shifted by two TRs (2.5 s each) to align the peak response (∼5 s) with behavioral events. The RSA involved the following steps: (i) extracting the BOLD signal within the right entorhinal cortex ROI from the preprocessed fMRI volumes without normalization or smoothing; (ii) calculating the mean orientation and speed for each fMRI volume based on the trajectory data; (iii) keeping only the volumes with movement speed in the top third; (iv) averaging volumes into bins of 5° orientation based on the mean orientation; (v) calculating the angular difference of the movement orientation between the volume bins; and (vi) calculating Fisher z-transformed Pearson correlations between the volume bins. We then compared the pattern similarity of pairs of volume bins offset by ±15° from the mod(*α*,60°) = 0° condition (aligned movements) with those offset by ±15° from the mod(*α*,60°) = 30 condition (misaligned movements). Pattern similarity was expected to be higher for aligned movements than for misaligned movements, thus reflecting GLRs. Two control analyses were performed. First, to ensure that the increase in pattern similarity was specific to 6-fold symmetry, the same analysis was performed for other types of rotational symmetry (4-, 5-, 7-, and 8-fold). Second, the analysis was also performed after excluding correlations that were ±15° from 0°, to ensure that increases in pattern similarity were not primarily driven by movements in the same direction, which could reflect head direction signals.

To investigate whether the magnitude of GLRs is modulated by the spatial environment (i.e., with or without spatial cues), we reconducted the GLR procedure in a secondary analysis, this time separating the data to calculate two distinct metrics for each testing day: one for Landmark PI and one for Pure PI. This isolated assessment enabled a comparison of GLR magnitude across the two navigational contexts.

### Statistical analysis

Our first statistical analysis examined the success of pharmacological intervention by comparing cortisol concentrations using an rANOVA with time and pharmacological intervention as within-subject factors. Because cortisol concentrations typically exhibit a right-skewed distribution, we conducted a natural log (ln) transformation to obtain normally distributed data. In case of violation of the sphericity assumption, we used Greenhouse-Geisser adjustment (and for the sake of presentation, we rounded the corrected degrees of freedom to the nearest whole number). Post-hoc pairwise comparisons were performed using Bonferroni-corrected *t*-tests (or Welch’s *t*-tests in case of unequal variances).

To test the hypothesis postulating differential effects of cortisol depending on subtask and incoming distance on PI performance, we then built a linear mixed model with PI performance (i.e., drop error) on the level of single trials as criterion, and subtask (two levels: Pure PI versus Landmark PI), incoming distance, and treatment (two levels: cortisol versus placebo) as within-subject predictors (Model 1; S1 Table).

To test the hypothesis concerning the effects of cortisol on the role of the landmark, we built three further linear mixed models (Models 2A–C; S1 Table). The first of these aimed at investigating the role of goal-to-landmark distance and its interaction with cortisol on PI performance and thus included PI performance on the level of single trials as criterion, goal-to-landmark distance (only in Landmark PI), and treatment as within-subject predictors. The second of these models aimed at investigating whether cortisol affected navigational pattern (irrespective of performance) and included movement-to-landmark distance (only in Landmark PI) on the level of single trials as criterion, and treatment as within-subject predictor. The third of these models aimed at investigating whether the navigational pattern (in interaction with cortisol) affected PI performance and thus included PI performance on the level of single trials as criterion, movement-to-landmark distance (only in Landmark PI), and treatment as within-subject predictors.

To evaluate the overall existence of GLRs, we conducted a one-tailed one-sample *t* test comparing activation of entorhinal cortex during aligned versus misaligned movements against zero (Model 3A; S1 Table). To further test the robustness of our findings, we performed a non-parametric permutation test. For each participant, we constructed a null distribution of similarity differences by randomly shuffling the angular labels and recomputing the similarity measure for each of 10,000 permutations. The position of the observed difference within this null distribution yielded a *p*-value, which we transformed into a signed *z*-statistic (positive and negative values allowed). At the group level, these *z*-statistics were tested against zero using a one-sample *t* test. To test whether GLRs were related to treatment, we conducted a linear model with GLRs as criterion, treatment and day as within-subject predictors, and age as covariate (Model 3B; S1 Table). For the secondary analysis, we conducted a linear model with GLRs as criterion, and treatment, day, and subtask as within-subject predictors, as well as age as covariate (Model 3C; S1 Table). We conducted separate *t*-tests for the control analyses (other symmetries or correction for heading direction). In the next step, to examine the behavioral relevance of GLRs, we built linear models with PI performance on the level of single trials as criterion, and subtask, incoming distance, and GLRs as within-subject predictors (Models 4A–B; S1 Table).

In all linear mixed models, “subject” was added as random factor, and age, testing day, and sequence (to control for order effects) as covariates. For all analyses, we centered within-subject parametric predictors (incoming distance, goal-to-landmark distance) to the participant’s mean and age to the grand mean of all participants [79]. For analysis of fixed effects, we always used type III sum of squares. In case of interactions between parametric predictors, we discretized all but one parametric predictor and calculated estimated marginal means (“emmeans” or “adjusted means”) or estimated marginal means of linear trends (“emtrends” or “conditional regression equations”) based on the minimum, mean, and maximum values of these discretized predictors. For post-hoc tests against zero or pairwise comparisons using Fisher’s tests, we used Šídák-adjustment correcting for number of discretized predictors (3), number of subtasks (2), number of treatments (2), or a combination of those. For an approximation of degrees of freedom, we used the Kenward-Roger method (and for the sake of presentation, we rounded them to the nearest whole number). To estimate effect sizes, we used Partial Eta-Squared (*η*_p_^2^) for *F*-tests and Cohen’s *d* for *t*-tests. Multicollinearity between predictors was not problematic (all variance inflation factors <5). Assumptions of statistical tests were verified before application, and all tests were conducted two-tailed (except for *t*-tests on GLRs) at a significance level of *α* = 0.05.

## Supporting information

S1 Text Analysis of integrated path and goal proximity representations. (PDF)

S1 Fig Overview of the virtual environment. **(A)** Locations of baskets (i.e., goal locations) and trees were equally distributed across a grid of 8 × 8 squares such that all participants visited all squares at least once in each subtask. Feedback was given according to the Euclidean distance between the response location and the correct goal location (i.e., drop error). In all subtasks, participants’ speed was linearly decreased to zero when their distance from the center of the arena was larger than 1.25*r vm. In Landmark PI, a landmark was located close to the center of the environment (at *x* = 1,600 vm, *y* = 800 vm). **(B)** Two examples of how locations could be assigned to the grid. Numbers represent the order at which the specific trees appeared. Pure PI: pure path integration, Landmark PI: landmark-supported path integration, r: radius, vm: virtual meters. Figure adapted from Bierbrauer and colleagues [22] under CC BY 4.0.
(EPS)

S2 Fig Predictors of PI performance and strategy use (extended plots). **(A)** The effect of longer incoming distances leading to higher drop errors was more pronounced when no spatial cues were available (left), and older age led to higher drop errors (right). **(B)** In Landmark PI, the movement distance to the landmark predicted performance, irrespective of treatment. Significant slopes are indicated via asterisks. Plots show estimated marginal means derived from linear mixed-effects models with an overlay of individual data points. Confidence bands represent SEM. CORT: cortisol, PLA: placebo, vm: virtual meters, n. s.: not significant, *** *p* < .001, ** *p* < .01.
(EPS)

S3 Fig Whole-brain analysis. Depicted are clusters with more than 5 voxels surviving an initial height threshold of *p* < .05, FWE-corrected for whole brain. For significant clusters, maximum probability tissue labels were derived from the Neuromorphometrics atlas in SPM. **(A)** Clusters in R precuneus, L precuneus, R middle occipital gyrus, L superior occipital gyrus, and L superior parietal lobule survived statistical correction. **(B)** No cluster survived statistical correction. **(C)** No cluster survived statistical correction LPI: landmark-supported path integration, PPI: pure path integration.
(EPS)

S4 Fig Masks used for ROIs. We used masks for the left and right pmEC (top-left panel), the left and right HC (top-right panel), the PC (bottom-left panel) and the left and right NC (bottom-right panel). PC: posterior cingulate, HC: hippocampus, NC: nucleus caudate, pmEC: posterior-medial entorhinal cortex.
(EPS)

S5 Fig Matched-difficulty analysis using a subsample of trials. **(A)** the whole sample, the performance difference between Pure PI (mean drop error: 2778.55) and Landmark PI (mean drop error: 1906.99) was substantial (*t*_(71)_ = −13.08, *p* < .001). **(B)** In the subsample including the easier half of Pure PI trials (mean drop error: 2319.30) and the more difficult half of Landmark PI trials (mean drop error: 2116.54), the difference was substantially smaller, even though it still slightly existed (*t*_(71)_ = −2.41, *p* = .018). (B) Posterior cingulate again showed higher activation for Landmark PI, while right caudate nucleus did not any longer (compared to full sample of trials). **(C)** When controlling for difficulty, caudate nucleus appears to be relevant in Landmark PI specifically under the influence of cortisol. Moreover, left hippocampus (but not right posterior-medial entorhinal cortex) showed higher activation in Pure PI under placebo. The data underlying this figure can be found at https://osf.io/c8u57.
(EPS)

S6 Fig Representation of integrated path. **(A)** Activation in ROIs scaling with instantaneous changes of integrated path, collapsed across subtasks and treatments. Activation in l CN, r CN, l pmEC, and r pmEC was significantly positively modulated by integrated path during the outgoing phase. **(B)** Subtask-specific parametric modulation by integrated path in ROIs during the outgoing phase (left) and during the incoming phase (right). Only ROIs showing significant modulation across subtasks and treatments during the outgoing phase (marked by the red rectangle) were tested for subtask-specific effects. No significant differences in parametric modulation effects emerged between subtasks. Bars depict mean beta estimates for the integrated path parametric regressor in each ROI. Error bars represent SEM. l: left, r: right, PC: posterior cingulate, HC: hippocampus, CN: caudate nucleus, pmEC: posterior-medial entorhinal cortex. *** *p* < .001, ** *p* < .01, * *p* < .05. The data underlying this figure can be found at https://osf.io/c8u57.
(EPS)

S7 Fig Representation of goal proximity. **(A)** Activation in ROIs scaling with instantaneous changes of goal proximity, collapsed across subtasks and treatments. Activation in l HC (during the incoming phase) and r HC (during both phases) was significantly positively modulated by goal proximity. Activation in l CN, r CN, and l pmEC (during the outgoing phase) was significantly negatively modulated by goal proximity. **(B)** Subtask-specific parametric modulation by goal proximity in ROIs during the outgoing phase (left) and during the incoming phase (right). Only ROIs showing significant modulation across subtasks and treatments during either phase (marked by the red rectangles) were tested for subtask-specific effects. No significant differences in parametric modulation effects emerged between subtasks. Bars depict mean beta estimates for the goal proximity parametric regressor in each ROI. Error bars represent SEM. l: left, r: right, PC: posterior cingulate, HC: hippocampus, CN: caudate nucleus, pmEC: posterior-medial entorhinal cortex. *** *p* < .001, ** *p* < .01, * *p* < .05. The data underlying this figure can be found at https://osf.io/c8u57.
(EPS)

S8 Fig Cortisol effects on GLRs and their behavioral relevance for the separate examination of Pure PI and Landmark PI. Note on Analysis: This figure presents data from a secondary analysis where GLRs were calculated separately for the Landmark PI and Pure PI subtasks. Consequently, each participant on each day has two distinct scores: one GLR for Landmark PI and one GLR for Pure PI. **(A)** Differences in GLR magnitude (i.e., pattern similarity difference) depending on treatment and day, controlling for the effect of subtask. **(B)** Differences in GLR magnitude depending on treatment and subtask on day one. **(C)** Regression between GLR magnitude and PI performance (drop error) on day one. Error bars and confidence bands represent SEM. GLR: grid-like representation, Pure PI: pure path integration, Landmark PI: landmark-supported path integration, CORT: cortisol, PLA: placebo, vm: virtual meters, n.s.: not significant, ** *p* < .01, * *p* < .05, † *p* < .10. The data underlying this figure can be found at https://osf.io/c8u57.
(EPS)

S9 Fig Parametric modulation of PI Performance. (A) Depicted are clusters with more than 10 voxels surviving an initial height threshold of *p* < .01, FDR-corrected for whole brain. The maximum probability tissue label for the significant cluster was derived from the Harvard–Oxford atlas in nilearn. Brain plots show color-coded *t*-values for the average contrast of drop error parametric modulation against baseline and for the average contrast between Landmark PI and Pure PI across participants. **(B)** Activation in the whole-brain cluster with significant positive modulation (increased activation with higher performance on a given trial; cluster 1 in Landmark PI and cluster 1 in Landmark PI > Pure PI) and negative modulation (reduced activation with higher performance in a given trial; cluster 1–3 in Pure PI) by inverted drop error (effects across treatments). Cluster indices correspond to brain regions listed in S5 Table. **(C)** FDR-correction was applied for the number of ROIs and number of contrasts at *α* < .01. No ROI showed significant modulation of the BOLD signal by (inverted) drop error on Pure PI or Landmark PI trials, nor for the difference between Landmark PI and Pure PI trials. ROIs were tested for significant parametric modulation across as well as between treatments. The contrast between Landmark PI and Pure PI was also not significant for any ROI. Bars depict mean beta estimates for the drop error parametric regressor in each cluster/ROI. Error bars represent SEM. l: left, r: right, PC: posterior cingulate, HC: hippocampus, CN: caudate nucleus, pmEC: posterior-medial entorhinal cortex, n.s.: not significant, ***p < .001, ** *p* < .01. The data underlying panels S9B and S9C can be found at https://osf.io/c8u57.
(EPS)

S10 Fig Effects of cortisol on PI subcomponents. **(A)** Cortisol (on trend) led to higher distance errors (left). In Landmark PI trials, a higher distance between goal location and landmark location predicted higher distance error and this effect did not differ between treatment conditions (right). **(B)** Cortisol (on trend) led to higher rotation errors (left). In Landmark PI trials, a higher distance between goal location and landmark location predicted higher rotation error and this effect did not differ between treatment conditions (right). Significant slopes are indicated via asterisks. Error bars and confidence bands represent SEM. CORT: cortisol, PLA: placebo, vm: virtual meters, n.s.: not significant, *** *p* < .001, ^†^ *p* < .10. The data underlying this figure can be found at https://osf.io/c8u57.
(EPS)

S1 Table Statistical models. Overview of statistical models used to examine effects of cortisol administration on PI (Model 1), navigational pattern in the presence of a landmark (Models 2A–2C), GLRs (Models 3A–3C), and to investigate the relationship between GLRs and PI (Models 4A–4B). Note that Models 3C and 4B were conducted in the secondary GLR analysis, which included separate GLR magnitudes for each subtask. PI: path integration, GLRs: grid-like representations (in right entorhinal cortex).
(PDF)

S2 Table Differences in cortisol concentrations after pharmacological treatment. To verify the success of cortisol administration, we analyzed cortisol concentrations using a repeated measures analysis of variance (rANOVA) with time point and pharmacological intervention as within-subject factors. We found significant main effects of treatment (*F*_(1,37)_ = 246.15, *p* < .001, *η*_p_^2^ = .869) and time point (*F*_(2,74)_ = 171.22, *p* < .001, *η*_p_^2^ = .822), and a significant time point × treatment interaction (*F*_(2,74)_ = 205.07, *p* < .001, *η*_p_^2^ = .847). Post-hoc pairwise comparisons revealed higher cortisol concentrations for both timepoints following cortisol compared to placebo administration (both *t* ≥ 17.3, both *p*_Bonferroni_ ≤ .001, both *d* ≥ 2.799), i.e., for the entire PI task, whereas no differences occurred at baseline (*t*_(37)_ = 0.55, *p*_Bonferroni_ = 1, *d* = 0.089). Cortisol concentrations represent mean ± standard deviation in nmol/l; *p*-values extracted from separate *t*-tests between treatments; *** *p* < .001.
(PDF)

S3 Table Separating behavioral effects for first and second half of the session. Effects of the separate models were mostly reflecting the full model, except for an only marginally significant effect of cortisol during the second half of the session. Only variables are shown that were significant in the main model. Other variables or effects did not show any change when separately considering two halves of the session.
(PDF)

S4 Table Global and local maxima of whole-brain analysis for contrasts. Brain regions exhibiting BOLD activations for individual contrasts. Reported are all clusters with more than 5 voxels, surviving an initial height threshold of *p* < 0.05, FWE-corrected for whole brain, as well as small volume corrected (SVC; FWE corrected *p* < 0.05) clusters for pmEC, HC, caudate nucleus, and PC/RSC. Clusters within ROIs are marked bold. For other significant clusters, maximum probability tissue labels are derived from the Neuromorphometrics atlas contained in SPM. L, left; R, right, *** *p* < .001, ** *p* < .01, * *p* < .05.
(PDF)

S5 Table Global and local maxima of whole-brain analyses for drop error parametric modulation. Brain regions showing stronger trial-wise BOLD modulation by (inverted) drop error on Landmark PI versus Pure PI trials; across treatments and for the contrast between treatments, respectively. Reported are all clusters with more than 10 voxels, surviving an initial height threshold of *p* < 0.01, FDR-corrected for whole brain. Maximum probability tissue labels are derived from the Harvard–Oxford atlas as implemented in nilearn. L, left; R, right, *** *p* < .001, ** *p* < .01.
(PDF)

S6 Table List of cognitive tests and questionnaires. Cognitive tests were only conducted on day one. These results are not part of this study.
(PDF)

## Abbreviations

ASHS: Automatic Segmentation of Hippocampal Subfields
BOLD: blood oxygenation level-dependent
CORT: cortisol
EC: entorhinal cortex
FDR: false discovery rate
fMRI: functional magnetic resonance imaging
FWE: family-wise error
GLM: general linear model
GRs: glucocorticoid receptors
GLRs: grid-like representations
HPA: hypothalamic–pituitary–adrenal axis
MRs: mineralocorticoid receptors
PC/RSC: posterior cingulate/retrosplenial cortex
PI: path integration
PLA: placebo
RSA: representational similarity analysis
ROI: region of interest; rANOVA, repeated measures analysis of variance
SAM: sympathetic–adrenal–medullary axis
TA: total acquisition time
