# Supplementary material for: Cortisol treatment impairs path integration and alters grid-like representations in the male human entorhinal cortex
Source: PLoS Biol. 2026 Mar 12;24(3):e3003661. doi: 10.1371/journal.pbio.3003661 (PMC12981458; doi:10.1371/journal.pbio.3003661)
Supplement: S1 Text — (PDF) [file pbio.3003661.s001.pdf]

S1 Text

Analysis of integrated path and goal proximity representations

Methods. To replicate the analyses of integrated path and goal proximity representations from Bierbrauer et al. (1), we built two further GLMs, each incorporating four regressors modeling the start phase, the outgoing phase, the incoming phase, and time periods of no movement, separately for each of the two subtasks (Landmark PI, Pure PI). In addition, each model included a parametric modulation regressor modeling the effect of integrated path (the cumulative distance traveled at each time point during the outgoing or incoming phase) or goal proximity (the Euclidean distance to the current goal at each time point during the outgoing or incoming phase), separately for movement periods of the outgoing and incoming phases of each subtask (resulting in 12 regressors per model). Regressors had separate onsets at the sampling rate of the behavioral data (every 200 ms) and a duration of 0 to model instantaneous changes in integrated path and goal proximity. Parametric modulators were normalized to values between 0 and 1 and mean-centered. All regressors were convolved with the canonical hemodynamic response function before entering the model. We included the same nuisance regressors and temporal filtering (cosine basis) as in the drop-error modulation GLM.

For each parametric modulator, and for each participant and treatment session (placebo, cortisol), we computed first-level contrast images for the phase- and subtask-specific parametric modulation effects against baseline (e.g., outgoing Landmark PI > baseline, incoming Pure PI > baseline), as well as phase-specific contrasts collapsed across subtasks (Landmark PI + Pure PI > baseline).

For the ROI analysis, we adapted the approach from Bierbrauer et al. (1) to our experimental design. Mean β values were extracted from the first-level contrast maps using seven a priori anatomical ROIs (PC, l HC, r HC, l CN, r CN, l pmEC, r pmEC; see also S4 Figure). For each parametric modulator, and separately for outgoing and incoming phases, we first averaged the subtask-collapsed contrast estimates across

treatment sessions within each participant and then tested mean β values against zero across participants using one-sample *t* tests. This step was done in all ROIs and both phases (7 ROIs × 2 phases = 14 tests), and the resulting *p* values were FDR-corrected at $\alpha < 0.05$.

Similar to Bierbrauer et al. (1), only if a phase-specific contrast against zero was significant after FDR correction in a given ROI, we proceeded to subtask-specific tests. For these ROIs and phases, we used the subtask-specific contrasts (Landmark PI and Pure PI) averaged across treatment sessions and (i) compared Landmark PI and Pure PI by means of paired *t* tests, and (ii) tested Landmark PI and Pure PI separately against zero using one-sample *t* tests. Within each of these two families of tests (Landmark PI vs. Pure PI; subtask vs. zero), *p* values were again FDR-corrected for the number of comparisons.

Moreover, we separately assessed treatment effects in all ROIs. For each parametric modulator, phase, and ROI, we compared the subtask-collapsed mean β values between placebo and cortisol sessions (CORT – PLA) using one-sample *t* tests across participants, with FDR correction across ROI × phase combinations. Lastly, we quantified the interaction between treatment and subtask by computing, for each participant, ROI, and phase, the subtask difference within each treatment and testing the interaction contrast "$(\text{Landmark PI} > \text{Pure PI})_{\text{CORT}} > (\text{Landmark PI} > \text{Pure PI})_{\text{PLA}}$" against zero using one-sample *t* tests; *p* values were FDR-corrected across ROIs and phases.

Results. For integrated path, we first tested whether BOLD activity in each ROI scaled with the parametric modulator when collapsing across subtasks (Landmark PI, Pure PI) and treatment sessions (S6 Figure). During the outgoing phase, we observed significant positive parametric effects in bilateral caudate nucleus (left CN: $t_{(34)} = 5.49$, $p_{\text{FDR}} < .0001$; right CN: $t_{(34)} = 4.79$, $p_{\text{FDR}} < .001$) and bilateral posterior-medial entorhinal cortex (left pmEC: $t_{(34)} = 4.18$, $p_{\text{FDR}} < .001$; right pmEC: $t_{(34)} = 5.17$, $p_{\text{FDR}} < .0001$). No other ROI showed a significant relationship with integrated path during the outgoing phase after FDR correction ($p_{\text{FDR}} > .05$), and no ROI showed a significant integrated-path effect

during the incoming phase (all $p_{FDR} > .05$). Next, we tested for subtask differences (Landmark PI vs Pure PI) in those ROIs showing an overall integrated-path effect during the outgoing phase (bilateral CN, bilateral pmEC). No Landmark PI–Pure PI difference survived FDR correction (all $p_{FDR} > .05$), indicating no subtask-specific modulation of integrated-path coding. However, both Landmark PI and Pure PI showed significant positive parametric modulation in bilateral caudate nucleus (left CN: Landmark PI $t_{(34)} = 5.02$, Pure PI $t_{(34)} = 5.28$, both $p_{FDR} < .001$; right CN: Landmark PI $t_{(34)} = 4.52$, Pure PI $t_{(34)} = 4.39$, both $p_{FDR} < .001$) and bilateral posterior-medial entorhinal cortex (left pmEC: Landmark PI $t_{(34)} = 4.22$, $p_{FDR} < .001$; Pure PI $t_{(34)} = 2.05$, $p_{FDR} < .05$; right pmEC: Landmark PI $t_{(34)} = 6.26$, $p_{FDR} < .0001$; Pure PI $t_{(34)} = 2.65$, $p_{FDR} < .05$), indicating robust integrated-path coding in these regions for both subtasks. We next tested whether cortisol affected the overall strength of integrated-path coding, collapsed across subtasks. No ROI showed a significant treatment effect for either phase after FDR correction (all $p_{FDR} > .05$). Finally, we tested whether cortisol differentially affected integrated-path coding in Landmark PI vs. Pure PI (treatment × subtask interaction). No ROI showed a significant interaction after FDR correction (all $p_{FDR} > .05$).

For goal proximity, we again first tested whether BOLD activity in each ROI scaled with the parametric modulator when collapsing across subtasks (Landmark PI, Pure PI) and treatment sessions (S7 Figure). During the incoming phase, we observed significant positive parametric effects in bilateral hippocampus (left HC: $t_{(34)} = 3.45$, $p_{FDR} < .01$; right HC: $t_{(34)} = 3.44$, $p_{FDR} < .01$). During the outgoing phase, right HC also showed a positive effect ($t_{(34)} = 4.72$, $p_{FDR} < .001$), whereas bilateral caudate nucleus and left posterior-medial entorhinal cortex showed significant negative parametric effects (left CN: $t_{(34)} = –3.62$, $p_{FDR} < .01$; right CN: $t_{(34)} = –2.92$, $p_{FDR} < .05$; left pmEC: $t_{(34)} = –3.95$, $p_{FDR} < .01$). No other ROI showed a significant relationship with goal proximity after FDR correction (all $p_{FDR} > .05$).

Next, we tested for subtask differences (Landmark PI vs Pure PI) in those ROI and phase combinations showing an overall goal-proximity effect (left HC incoming; right HC

outgoing and incoming; left and right CN outgoing; left pmEC outgoing). No Landmark PI–Pure PI difference survived FDR correction in any of these regions (all $p_{FDR} > .05$), indicating no subtask-specific modulation of goal-proximity coding.

We then examined whether the goal-proximity signal in these ROIs was reliably present within each subtask. Both Landmark PI and Pure PI showed significant positive parametric modulation in the hippocampus (left HC incoming: Landmark PI $t_{(34)} = 2.78$, $p_{FDR} < .05$; Pure PI $t_{(34)} = 3.19$, $p_{FDR} < .01$; right HC outgoing: Landmark PI $t_{(34)} = 3.17$, Pure PI $t_{(34)} = 3.99$, both $p_{FDR} < .01$; right HC incoming: Landmark PI $t_{(34)} = 2.95$, $p_{FDR} < .05$; Pure PI $t_{(34)} = 2.60$, $p_{FDR} < .05$). In contrast, both subtasks showed significant negative goal-proximity modulation in bilateral caudate nucleus during the outgoing phase (left CN: Landmark PI $t_{(34)} = –3.00$, Pure PI $t_{(34)} = –2.94$, both $p_{FDR} < .05$; right CN: Landmark PI $t_{(34)} = –2.69$, $p_{FDR} < .05$; Pure PI $t_{(34)} = –2.23$, $p_{FDR} < .05$) and in left posterior-medial entorhinal cortex (Landmark PI $t_{(34)} = –3.52$, $p_{FDR} < .01$; Pure PI $t_{(34)} = –2.68$, $p_{FDR} < .05$). Thus, across subtasks, the hippocampus consistently showed stronger activation with increasing goal proximity, whereas caudate nucleus and left posterior-medial entorhinal cortex showed the opposite pattern.

We next tested whether cortisol affected the overall strength of goal-proximity coding, collapsed across subtasks. No ROI showed a significant treatment effect for either phase after FDR correction (all $p_{FDR} > .05$). Finally, we tested whether cortisol differentially affected goal-proximity coding in Landmark PI versus Pure PI (treatment × subtask interaction). No ROI showed a significant interaction after FDR correction (all $p_{FDR} > .05$).

Our results are only partly in line with those reported by Bierbrauer et al. (1). Specifically, their study found significant deactivation with increasing integrated path in bilateral entorhinal cortex and bilateral hippocampus during the outgoing phase and significant activation with increasing integrated path in the same ROIs during the incoming phase. In contrast, we observed integrated path-related activation in left and right posterior-medial entorhinal cortex, in addition to activation in left and right caudate nucleus (which Bierbrauer et al. (1) did not include in their ROI analyses), during the outgoing phase,

but no signal modulation by integrated path in any ROI during the incoming phase. Moreover, while Bierbrauer et al. (1) found subtask-specific modulation by integrated path in entorhinal cortex and hippocampus only for the Pure PI condition, in the present study left and right caudate nucleus and left and right posterior-medial entorhinal cortex activation was modulated by integrated path across both Pure PI and Landmark PI during the outgoing phase. Regarding goal proximity representations, Bierbrauer et al. (1) reported bilateral hippocampus activation increasing with goal proximity during both outgoing and incoming phases, and bilateral entorhinal cortex activation increasing with goal proximity during the outgoing phase. We similarly found increased activation with higher goal proximity in right hippocampus during both phases and in left hippocampus during the incoming phase, but deactivation with higher goal proximity in left posterior-medial entorhinal cortex as well as left and right caudate nucleus (which Bierbrauer et al. (1) did not include in their ROI analyses) during the outgoing phase. We found no evidence for subtask-specific modulation by goal-proximity, while Bierbrauer et al. (1) did not report any subtask-specific contrasts for this measure.

While in principle, our finding of integrated path and goal proximity representations in posterior-medial entorhinal cortex (and additionally, caudate nucleus) as well as goal proximity representations in hippocampus, are in line with the results reported by Bierbrauer et al. (1), some deviations emerged regarding the phase- and subtask-specificity of these effects. Several differences between both studies' sample characteristics, design, and fMRI analysis approach could be related to these deviations. Specifically, Bierbrauer et al. (1) had a single-session fMRI experiment including equal numbers of male and female participants while the current study involved a two-day crossover with pharmacological manipulation, including only male participants. The Apple Game used in the previous study involved three subtask types (Pure PI, Boundary PI, Landmark PI), while the Boundary PI condition was not included in our experiment, potentially altering integrated path and goal proximity coding patterns. Moreover, using a shim box around the MTL in our fMRI experiment potentially reduces comparability of

affected ROI data. Importantly, Bierbrauer et al. (1) applied FreeSurfer-derived ROI masks in native space, while we performed ROI analyses using atlas-derived masks on MNI-normalized data. In addition, Bierbrauer et al. (1) tested the entire entorhinal cortex, while our analyses of integrated path and goal proximity representations focused on left and right posteromedial subsections, which is likely to have influenced effects seen in this ROI. Lastly, differences in fMRI data modeling could account for the deviations between both studies, including a smaller number of regressors in the present study's parametric modulation GLMs (12 vs. 24 regressors) and slight differences in the inclusion of nuisance regressors.

References

1. Bierbrauer A, Kunz L, Gomes CA, Luhmann M, Deuker L, Getzmann S et al. Unmasking selective path integration deficits in Alzheimer's disease risk carriers. Sci. Adv. 2020; 6(35):eaba1394.
