# Supplementary material for: Cortisol treatment impairs path integration and alters grid-like representations in the male human entorhinal cortex
Source: PLoS Biol. 2026 Mar 12;24(3):e3003661. doi: 10.1371/journal.pbio.3003661 (PMC12981458; doi:10.1371/journal.pbio.3003661)
Supplement: S1 Table — Overview of statistical models used to examine effects of cortisol administration on PI (Model 1), navigational pattern in the presence of a landmark (Models 2A–2C), GLRs (Models 3A–3C), and to investigate the relationship between GLRs and PI (Models 4A–4B). Note that Models 3C and 4B were conducted in the secondary GLR analysis, which included separate GLR magnitudes for each subtask. PI: path integration, GLRs: grid-like representations (in right entorhinal cortex). (PDF) [file pbio.3003661.s012.pdf]

S1 Table. Statistical Models.

Model	Criterion	Predictors (within subject)	Predictors (between subjects)	Covariates	Random Effect
**Effects of Subtask, Path Distance and Cortisol on PI Performance**					
**1**	Drop Error	Subtask, Incoming Distance, Treatment	-	Age, Day, Sequence	Subject
**Effects of Landmark (only Landmark PI trials)**					
**2A**	Drop Error	Goal-to-Landmark Distance, Treatment	-	Age, Day, Sequence	Subject
**2B**	Movement-to-Landmark Distance	Treatment	-	Age, Day, Sequence	Subject
**2C**	Drop Error	Movement-to-Landmark Distance, Treatment		Age, Day, Sequence	Subject
**Effects of cortisol on GLRs**					
**3A**	GLRs	-	-	-	-
**3B**	GLRs	Treatment, Day	-	Age	-
**3C**	GLRs	Treatment, Day, Subtask		Age	-
**Effects of Subtask, Path Distance, Cortisol and GLRs on PI Performance**					
**4A**	Drop Error	Subtask, Treatment, Incoming Distance	GLRs	Age	-
**4B**	Drop Error	Subtask, Treatment, Incoming Distance	GLRs	Age	-

Note. Overview of statistical models used to examine effects of cortisol administration on PI (Model 1), navigational pattern in the presence of a landmark (Models 2A–2C), GLRs (Models 3A–C), and to investigate the relationship between GLRs and PI (Models 4A-B). Note that Models 3C and 4B were conducted in the secondary GLR analysis, which included separate GLR magnitudes for each subtask. PI: path integration, GLRs: grid-like representations (in right EC).
