# Supplementary material for: Cortisol treatment impairs path integration and alters grid-like representations in the male human entorhinal cortex
Source: PLoS Biol. 2026 Mar 12;24(3):e3003661. doi: 10.1371/journal.pbio.3003661 (PMC12981458; doi:10.1371/journal.pbio.3003661)
Supplement: S2 Table — To verify the success of cortisol administration, we analyzed cortisol concentrations using a repeated measures analysis of variance (rANOVA) with time point and pharmacological intervention as within-subject factors. We found significant main effects of treatment (F(1,37) = 246.15, p < .001, ηp2 = .869) and time point (F(2,74) = 171.22, p < .001, ηp2 = .822), and a significant time point × treatment interaction (F(2,74) = 205.07, p < .001, ηp2 = .847). Post-hoc pairwise comparisons revealed higher cortisol concentrations for both timepoints following cortisol compared to placebo administration (both t ≥ 17.3, both pBonferroni ≤ .001, both d ≥ 2.799), i.e., for the entire PI task, whereas no differences occurred at baseline (t(37) = 0.55, pBonferroni = 1, d = 0.089). Cortisol concentrations represent mean ± standard deviation in nmol/l; p-values extracted from separate t-tests between treatments; *** p < .001. (PDF) [file pbio.3003661.s013.pdf]

S2 Table. Differences in cortisol concentrations after pharmacological treatment.

timepoint	placebo (*n* = 39)	cortisol (*n* = 39)
baseline	3.55 ± 2.72	3.73 ± 2.60
+30 mins	2.80 ± 2.45	323.44 ± 338.44***
+110 mins	1.66 ± 1.05	57.54 ± 82.56***

Note. To verify the success of cortisol administration, we analyzed cortisol concentrations using a repeated-measures analysis of variance (rANOVA) with timepoint and pharmacological intervention as within-subject factors. We found significant main effects of treatment ($F_{(1,37)}$ = 246.15, $p < .001$, $\eta_p^2 = .869$) and timepoint ($F_{(2,74)}$ = 171.22, $p < .001$, $\eta_p^2 = .822$), and a significant timepoint x treatment interaction ($F_{(2,74)}$ = 205.07, $p < .001$, $\eta_p^2 = .847$). Post-hoc pairwise comparisons revealed higher cortisol concentrations for both timepoints following cortisol compared to placebo administration (both $t \geq 17.3$, both $p_{Bonferroni} \leq .001$, both $d \geq 2.799$), i.e., for the entire PI task, whereas no differences occurred at baseline ($t_{(37)}$ = 0.55, $p_{Bonferroni}$ = 1, d = 0.089). Cortisol concentrations represent mean ± standard deviation in nmol/l; *p*-values extracted from separate *t*-tests between treatments; *** $p < .001$.
