# Supplementary material for: Cortisol treatment impairs path integration and alters grid-like representations in the male human entorhinal cortex
Source: PLoS Biol. 2026 Mar 12;24(3):e3003661. doi: 10.1371/journal.pbio.3003661 (PMC12981458; doi:10.1371/journal.pbio.3003661)
Supplement: S3 Table — Effects of the separate models were mostly reflecting the full model, except for an only marginally significant effect of cortisol during the second half of the session. Only variables are shown that were significant in the main model. Other variables or effects did not show any change when separately considering two halves of the session. (PDF) [file pbio.3003661.s014.pdf]

S3 Table. Separating behavioral effects for first and second half of the session.

variable	full model		first half		second half	
	F	*p*	*F*	*p*	*F*	*p*
subtask	432.88	< .001	216.28	< .001	218.10	< .001
**treatment**	**10.46**	**.001**	**7.58**	**.006**	**3.16**	**.075**
incoming distance	364.99	< .001	145.06	< .001	222.57	< .001
age	12.23	.001	14.23	< .001	8.76	.005
subtask x incoming distance	44.99	< .001	16.58	< .001	28.84	< .001

Note. Effects of the separate models were mostly reflecting the full model, except for an only marginally significant effect of cortisol during the second half of the session. Only variables are shown that were significant in the main model. Other variables or effects did not show any change when separately considering two halves of the session.
