# Supplementary material for: Cortisol treatment impairs path integration and alters grid-like representations in the male human entorhinal cortex
Source: PLoS Biol. 2026 Mar 12;24(3):e3003661. doi: 10.1371/journal.pbio.3003661 (PMC12981458; doi:10.1371/journal.pbio.3003661)
Supplement: S4 Table — Brain regions exhibiting BOLD activations for individual contrasts. Reported are all clusters with more than 5 voxels, surviving an initial height threshold of p < 0.05, FWE-corrected for whole brain, as well as small volume corrected (SVC; FWE corrected p < 0.05) clusters for pmEC, HC, caudate nucleus, and PC/RSC. Clusters within ROIs are marked bold. For other significant clusters, maximum probability tissue labels are derived from the Neuromorphometrics atlas contained in SPM. L, left; R, right, *** p < .001, ** p < .01, * p < .05. (PDF) [file pbio.3003661.s015.pdf]

S4 Table. Global and local maxima of whole brain analysis for contrasts.

Contrast / Region	Voxels	X	Y	Z	*t*-score
**Landmark PI > Pure PI**					
R precuneus	125	18	-58	18	9.11***
L precuneus	541	-2	-52	52	8.03***
L precuneus	58	-8	-62	18	7.65***
R middle occipital gyrus	20	38	-78	35	6.85***
L superior occipital gyrus	33	-25	-80	32	6.73**
L superior parietal lobule	5	-20	78	45	6.33*
**Posterior cingulate (SVC)**	**233**	**8**	**-50**	**5**	**6.65*** **
**R nucleus caudate (SVC)**	**29**	**15**	**2**	**20**	**4.45***
**CORT > PLA**			No significant clusters		
**$(\text{Landmark PI > Pure PI})_{CORT}$ > $(\text{Landmark PI > Pure PI})_{PLA}$**					
**R pmEC (SVC)**	**4**	**22**	**-20**	**-28**	**2.78***
**R nucleus caudate (SVC)**	**177**	**10**	**8**	**12**	**4.62** **
**L nucleus caudate (SVC)**	**156**	**-12**	**-5**	**20**	**3.90** **
**Pure PI > Landmark PI**			No significant clusters		
**PLA > CORT**			No significant clusters		
**$(\text{Landmark PI > Pure PI})_{PLA}$ > $(\text{Landmark PI > Pure PI})_{CORT}$**			No significant clusters		

Note. Brain regions exhibiting BOLD activations for individual contrasts. Reported are all clusters with more than 5 voxels, surviving an initial height threshold of $p < 0.05$, FWE-corrected for whole brain, as well as small volume corrected (SVC; FWE corrected $p < 0.05$) clusters for pmEC, HC, caudate nucleus, and PC/RSC. Clusters within ROIs are marked bold. For other significant clusters, maximum probability tissue labels are derived from the Neuromorphometrics atlas contained in SPM. L, left; R, right, *** $p < .001$, ** $p < .01$, * $p < .05$.
