# Supplementary material for: Cortisol treatment impairs path integration and alters grid-like representations in the male human entorhinal cortex
Source: PLoS Biol. 2026 Mar 12;24(3):e3003661. doi: 10.1371/journal.pbio.3003661 (PMC12981458; doi:10.1371/journal.pbio.3003661)
Supplement: S5 Table — Brain regions showing stronger trial-wise BOLD modulation by (inverted) drop error on Landmark PI versus Pure PI trials; across treatments and for the contrast between treatments, respectively. Reported are all clusters with more than 10 voxels, surviving an initial height threshold of p < 0.01, FDR-corrected for whole brain. Maximum probability tissue labels are derived from the Harvard–Oxford atlas as implemented in nilearn. L, left; R, right, *** p < .001, ** p < .01. (PDF) [file pbio.3003661.s016.pdf]

S5 Table. Global and local maxima of whole brain analyses for drop error parametric modulation.

Cluster No.	Region	Voxels	X	Y	Z	*z*-score
**Landmark PI (across treatments)**						
1	L Inferior Occipital Lobule	10	-18	-98	-3	6.03**
**Landmark PI (CORT > PLA)**						
-						
**Pure PI (across treatments)**						
1	R Cerebellum	65	15	-63	-18	-8.15***
2	L Middle Occipital Lobule	53	-18	-93	0	-7.94**
3	Outside of defined BAs	58	18	-43	20	-5.19**
**Pure PI (CORT > PLA)**						
-						
**Landmark PI > Pure PI (across treatments)**						
1	L Inferior Occipital Lobule	29	-18	-93	0	8.24**
**Landmark PI > Pure PI (CORT > PLA)**						
-						

Note. Brain regions showing stronger trial-wise BOLD modulation by (inverted) drop error on Landmark PI versus Pure PI trials; across treatments and for the contrast between treatments, respectively. Reported are all clusters with more than 10 voxels, surviving an initial height threshold of $p < 0.01$, FDR-corrected for whole brain. Maximum probability tissue labels are derived from the Harvard-Oxford atlas as implemented in nilearn. L, left; R, right, *** $p < .001$, ** $p < .01$.
