# Supplementary material for: Cortisol treatment impairs path integration and alters grid-like representations in the male human entorhinal cortex
Source: PLoS Biol. 2026 Mar 12;24(3):e3003661. doi: 10.1371/journal.pbio.3003661 (PMC12981458; doi:10.1371/journal.pbio.3003661)
Supplement: S6 Table — Cognitive tests were only conducted on day one. These results are not part of this study. (PDF) [file pbio.3003661.s017.pdf]

S6 Table. List of cognitive tests and questionnaires.

Cognitive test	Reference	Cognitive dimension
Ray-Figure Task	Rey (1)	Visual memory
Logical memory	Wechsler (2)	Verbal memory
Digit span test	Wechsler (2)	Verbal working memory
Block span test	Corsi (3)	Visual working memory
Trailmaking A & B	Reitan (4)	Visual attention and Task switching
**Questionnaire**	**Reference**	**Measured variable**
Positive and Negative Affect Schedule	Krohne et al. (5)	Positive and negative affect
Edinburgh Handedness Inventory	Oldfield (6)	Laterality quotient
Navigation Strategy Questionnaire	Zhong and Kozhevnikov (7)	Navigational strategy preferences
Life Threatening Experiences	Brugha and Cragg (8)	Traumatic experiences in the last 12 months
Big Five Inventory K	Rammstedt and John (9)	Big five personality dimensions
Depression-Anxiety-Stress Scale	Nilges and Essau (10)	Depression, anxiety, and stress levels
Apple Game Questionnaire	-	Strategy use during Apple Game

Note. Cognitive tests were only conducted on day one. These results are not part of this study.

References

1. Rey A. L'examen psychologique dans les cas d'encéphalopathie traumatique. (Les problems.). [The psychological examination in cases of traumatic encepholopathy. Problems.]. Archives de Psychologie 1941; 28:215–85.

2. Wechsler D. Wechsler Adult Intelligence Scale–Fourth edition: Technical and interpretive manual. San Antonio, TX: Pearson Assessment; 2008.

3. Corsi PM. Human memory and the medial temporal region of the brain. In: ; 1972 Available from: URL: https://api.semanticscholar.org/CorpusID:140928756.

4. Reitan RM. The relation of the trail making test to organic brain damage. J Consult Psychol 1955; 19(5):393–4.

5. Krohne HW, Egloff B, Kohlmann C-W, Tausch A. Untersuchungen mit einer deutschen Version der "Positive and Negative Affect Schedule" (PANAS). [Investigations with a German version of the Positive and Negative Affect Schedule (PANAS).]. Diagnostica 1996; 42(2):139–56.

6. Oldfield RC. The assessment and analysis of handedness: The Edinburgh inventory. Neuropsychologia 1971; 9(1):97–113.

7. Zhong JY, Kozhevnikov M. Relating allocentric and egocentric survey-based representations to the self-reported use of a navigation strategy of egocentric spatial updating. Journal of Environmental Psychology 2016; 46:154–75. Available from: URL: http://www.sciencedirect.com/science/article/pii/S0272494416300263.

8. Brugha TS, Cragg D. The List of Threatening Experiences: the reliability and validity of a brief life events questionnaire. Acta Psychiatr Scand 1990; 82(1):77–81.

9. Rammstedt B, John OP. Kurzversion des Big Five Inventory (BFI-K). Diagnostica 2005; 51(4):195–206.

10. Nilges P, Essau C. Die Depressions-Angst-Stress-Skalen: Der DASS--ein Screeningverfahren nicht nur für Schmerzpatienten. Schmerz 2015; 29(6):649–57. Available from: URL: https://link.springer.com/article/10.1007/s00482-015-0019-z.
